# Therapeutic interventions for acute complete ruptures of the ulnar collateral ligament of the thumb: a systematic review

**DOI:** 10.12688/f1000research.15065.1

**Published:** 2018-06-08

**Authors:** Mark Mikhail, Justin C. R. Wormald, Neal Thurley, Nicholas Riley, Benjamin J. F. Dean

**Affiliations:** 1 Department of Plastic, Reconstructive and Hand Surgery, John Radcliffe Hospital, Oxford, OX3 9DU, UK; 2 Nuffield Department of Orthopaedics, Rheumatology and Musculoskeletal Sciences (NDORMS), Botnar Research Centre, University of Oxford, Oxford, OX3 7LD, UK; 3 Department of Plastic, Reconstructive and Burns Surgery, Stoke Mandeville Hospital, Aylesbury, HP21 8AL, UK; 4 Bodleian Health Care Libraries, Cairns Library, John Radcliffe Hospital, Oxford, OX3 9DU, UK; 5 Nuffield Orthopaedic Centre, Oxford University Hospitals NHS Foundation Trust, Oxford, OX3 7LD, UK

**Keywords:** ulnar collateral ligament; thumb; rupture; surgery

## Abstract

**Background: **The aim of this study was to evaluate the effectiveness of interventions for acute complete rupture of the ulnar collateral ligament (UCL) of the thumb in adults.

**Methods: **The following databases were searched: MEDLINE and EMBASE via OVID, CINAHL and SPORTDiscus via EBSCO, from database inception to 31 ^st^ January 2018. Inclusion criteria were: (i) randomised controlled clinical trials (RCTs) or study of intervention with a comparator; (ii) participants with diagnosis of acute complete rupture of the UCL of the thumb; (iii) participants aged 18 years of age or older at enrolment; and (iv) published in a peer-reviewed English-language journal.

**Results: **In total, six studies were identified for inclusion after screening. All studies had a high risk of bias. Three studies were retrospective comparative case series which compared two different surgical techniques (bone anchor versus pull out suture, suture versus pull out suture, suture versus steel wire). Of these studies, three were RCTs, two of which compared different rehabilitation regimes in patients managed surgically (plaster versus early mobilization, new spica versus standard spica). The remaining RCT compared two different rehabilitation regimes in a mixed group of surgically/non-surgically treated patients. The RCT comparing a standard spica with a new spica demonstrated a statistically significant improvement in outcomes with the new spica at all time points (range of motion, Dreiser index and VAS); this was also the only study to provide sufficient outcome data for further analysis.

**Conclusion: **There is no prospective evidence comparing surgery to non-operative treatment for acute complete ruptures of the ulnar collateral ligament of the thumb. There is weak evidence to suggest that early mobilisation may be beneficial following surgical repair. Further research is necessary to better define which patients benefit from which specific interventions.

## Introduction

Acute complete ruptures of the ulnar collateral ligament (UCL) of the thumb are common injuries, accounting for around 50 in 100,000 presentations to Accident and Emergency departments. There is controversy as how to manage complete ruptures of the UCL best, although there is a degree of consensus regarding the broader treatment algorithm and general agreement that ‘true’ Stener lesions should be managed operatively ^1, 2^. The rate of the Stener lesion varies widely in the literature, perhaps reflecting the lack of reliability and accuracy of the various methods of diagnosis ^3, 4^. 

Patients should be assessed clinically to determine the degree of instability of the metacarpophalangeal joint (MCPJ) in both extension and 30° of flexion to test both proper and accessory collateral ligaments ^5^. There is some evidence to suggest that the greater the instability the higher the chances are that a Stener lesion is present ^6, 7^. While there is evidence to support both the use of ultrasound and MRI, the latter appears slightly superior in terms of sensitivity and specificity ^8– 10^. A recent study by Stoop *et al.* investigated which factors predict the chances of surgery in UCL injuries ^11^. It was found that not only did patient characteristics influence the chances of surgery, but that the individual surgeon’s preference was also predictive.

Our aim was to perform a systematic review of the effectiveness of available interventions for acute complete rupture of the ulnar collateral ligament of the thumb in terms of patient-reported outcome measures and to assess the rates of adverse outcomes associated with these interventions.

## Methods

The systematic review was developed in accordance with the PRISMA statement ( Supplementary File 1 contains a completed PRISMA checklist), using methodology decribed in the Cochrane Handbook for Systematic Reviews of Interventions. The protocol was developed prospectively and peer reviewed locally before registration on the PROSPERO database ( CRD42018087656).

### Data sources and searches

A comprehensive search strategy was created in collaboration with a research librarian (N.T.) and was designed to capture all relevant articles pertaining to inventions for acute complete ruptures of the ulnar collateral ligament of the thumb ( Supplementary File 2). The full search strategy is detailed on the PROSPERO website. The search strategy was applied to the following bibliographic databases from database inception until 31 ^st^ January 2018: MEDLINE and EMBASE via OVID, CINAHL and SPORTDiscus via EBSCO from database inception until 31 ^st^ January 2018.

### Inclusion/exclusion criteria

The inclusion and exclusion criteria were defined prospectively during the protocol stage. Any study relating to acute complete ruptures of the ulnar collateral ligament of the thumb MCPJ in adults was included. Studies had to contain an intervention and a comparator (i.e. both non-randomised controlled trials, and randomised controlled trials, including semi/quasi randomised, cluster randomised trials and comparative case series). Any therapeutic intervention or control treatments were included.

### Selection of studies

Duplicates were removed and relevant studies identified from the search were imported into Covidence for screening. Studies were independently screened by title and abstract by two authors (B.J.F.D. and M.M.). This was followed by a full-text evaluation of the selected studies from the first selection step these authors. Disagreement between the two reviewers was solved by consensus involving a third author (J.C.R.W.).

### Data extraction

Two reviewers (M.M. and B.J.F.D) independently extracted data. Data was extracted using a custom data extraction sheet in Covidence. Any inconsistencies between the two reviewers’ forms were resolved by consensus discussion. A third review (J.C.R.W.) was available for any disagreement that could not be resolved by this initial discussion.

If data was not available from full-text articles or trial registrations, authors were contacted to provide this information. If authors were not contactable as regards additional data, then this aspect of the study was excluded from the data synthesis. If contactable authors did not respond to initial requests, they were sent two subsequent reminders over a minimum of 6 weeks. If there was still no response for the additional data, then this aspect of the study was excluded from the data synthesis.

### Risk of bias assessment

Included studies were assessed for risk of bias by two independent raters (B.J.F.D. and M.M.) using the Cochrane Collaboration’s tool for assessing risk of bias in randomised trials ^12^. This followed the description in the Cochrane Handbook for Systematic Review of Interventions, version 5.1 (Part 2: 8.5.1) ^12^. Any disagreements between ratings were resolved by discussion between the raters. A third party (J.C.R.W.) was available in any case where disagreements persisted after discussion.

### Data analysis

Descriptive analysis was performed for all demographic, intervention and outcome data to facilitate narrative interpretation and comparison across studies. It was decided that a direct-comparison meta-analysis would only be performed if data was available for similar time-points, outcomes and interventions across two or more studies. As this was not possible with the identified studies, we conducted a narrative synthesis of the results based on the domains of interest.

## Results

**Figure 1.**
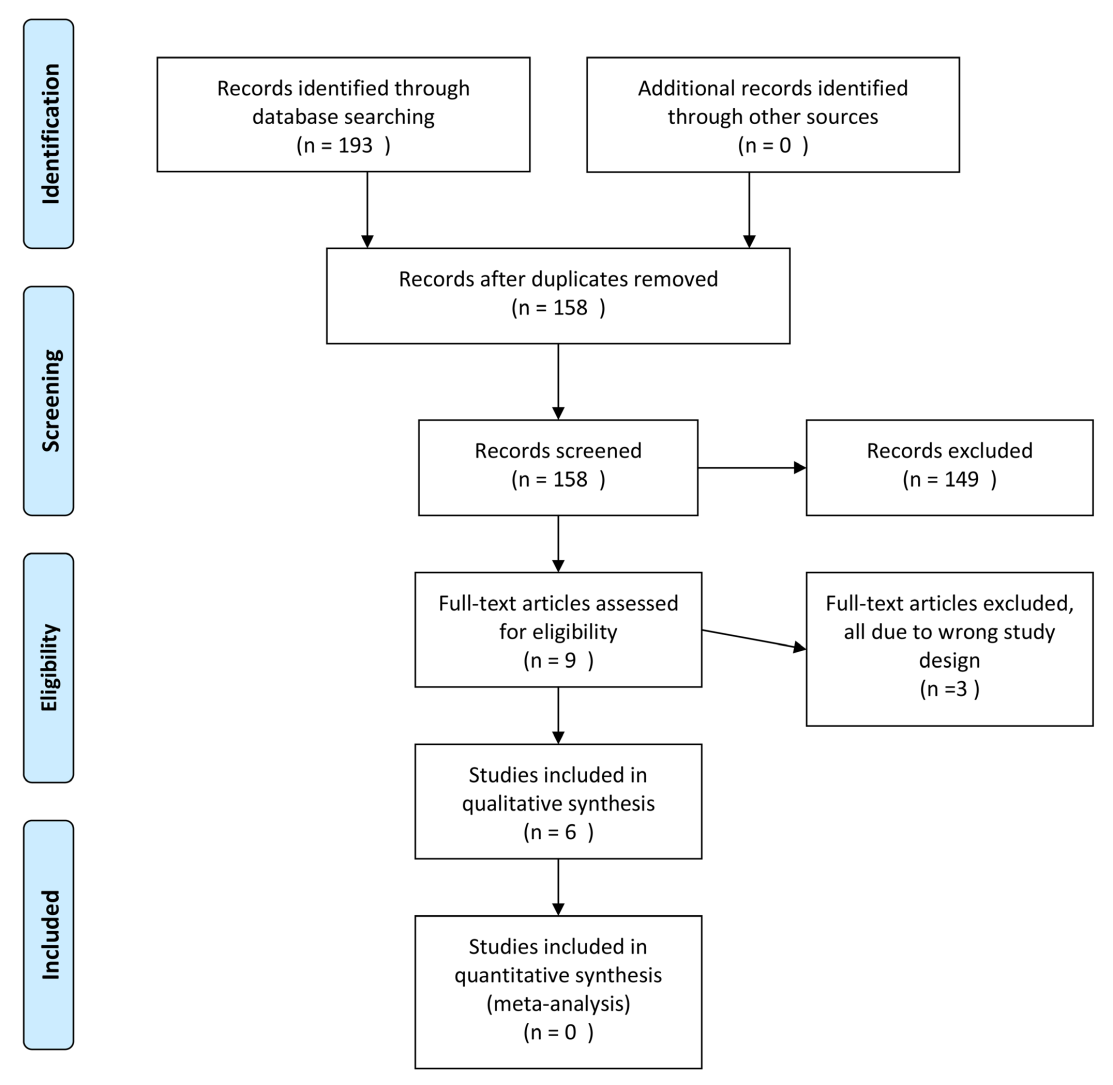
PRISMA flow diagram.

A total of 158 studies were identified by the search, after duplicates were removed. After screening by full-text, six studies were identified as eligible for inclusion ( Figure 1). Of these, three were randomized controlled trials (RCTs), and three were retrospective comparative case series. The number of studies identified and excluded at each stage is detailed in Figure 1.

Study characteristics of the included trials including the interventions and comparators are provided in Table 1. Of the three randomised controlled trials, two assessed the outcomes of different rehabilitation regimes in patients who had been exclusively treated with surgery ^13, 14^. The remaining RCT assessed the outcome in patients managed both surgically and non-surgically, who were randomised to treatment with either a plaster cast or a functional splint ^15^. All three retrospective comparative case series compared different surgical techniques in patients exclusively managed surgically ^16– 18^. Table 2 details the basic demographics of the intervention and comparator groups, as well as the details about the outcome data provided. The full details of all included studies and the forest plots are included within the supplementary material ( Supplementary File 3– Supplementary File 15).

**Table 1.** Study characteristics.

Author	Year	Journal	Setting	Population	Type of study	Intervention	Comparator	Primary outcome	Outcomes	Time points
Crowley *et al.* ^13^	2013	Techniques in Hand and Upper Extremity Surgery	Hospital plastic surgery department	Acute complete UCL ruptures repaired with Mitek anchors	Randomised controlled trial	Early active mobilisation	Plaster immobilisation	None specified	Range of motion, return to work, normal hand function, complications	1 month, 3 months, 6 months
Katolik *et al.* ^16^	2008	Plastic and Reconstructive Surgery	Hand Surgery Unit	Acute complete UCL ruptures treated with surgery	Retrospective comparative case series	Bone anchor repair	Pull out suture repair	None specified	Range of motion, Pinch strength, patient satisfaction, complication	Final follow up
Lane ^17^	1991	American Journal of Sports Medicine	Orthopaedic Surgery Department	Acute complete UCL ruptures treated with surgery	Retrospective comparative case series	Suture repair (’new method’)	Pull out suture and K wire stabilisation of MCPJ	None specified	Range of motion, strength (full vs partial), overall outcome (excellent vs good), complications	Final follow up
Rocchi *et al.* ^14^	2014	European Journal of Physical and Rehabiliation Medicine	Orthopaedic Hand Surgery Department	Acute complete UCL ruptures treated with surgery	Randomised Controlled Trial	New spica	Standard spica	None specified	Range of motion, Dreiser index, VAS, Tip pinch strength, Complications	1 month, 2 months, 6 months, 12 months
Saetta *et al.* ^18^	1992	Journal of Hand Surgery – British volume	Accident and Emergency Department	Acute complete UCL ruptures treated with surgery	Retrospective comparative case series	Suture repair	Steel wire repair	None specified	Key strength, Pinch strength, Grasp strength, Functional result (excellent vs not)	Final follow up
Sollerman *et al.* ^15^	1991	Acta Orthopaedica Scandinavica	Hand Surgery Department	Acute UCL ruptures treated surgically/ non surgically	Randomised Controlled Trial	Functional splint	Plaster cast	None specified	Range of motion, Pinch grip strength, Sick leave	Final follow up

UCL, ulnar collateral ligament; MCPJ, metacarpophalangeal joint.

**Table 2.** Details of study participants demographics, inclusion/exclusion criteria and whether data was provided.

Author	Year	Inclusion criteria	Exclusion criteria	Intervention group age, years	Comparator group age, years	Intervention group sex	Comparator group sex	Data comments
Crowley *et al.* ^13^	2013	All patients undergoing surgery for UCL rupture – diagnostic criteria not specified	K wire used in surgery	26 (range 20–43)	50 (range 37–72)	4 male, 2 female	4 male, 2 female	All data other than complication rate not available according to author response
Katolik *et al.* ^16^	2008	<4 weeks old, laxity >30° in 30° flexion or >10 increased laxity compared to contralateral side	Avulsion fractures >10% of joint surface	32	32	Not reported	Not reported	All data other than complication rate not fully reported and author responded to confirm not available
Lane ^17^	1991	Grade 3 UCL ruptures – >35° laxity in 30° flexion or >15° laxity relative to contralateral side		Not reported	Not reported	Not reported	Not reported	Outcome data complete and unable to contact author for full demographic data
Rocchi *et al.* ^14^	2014	>30° laxity or >20° laxity relative to contralateral side	Partial tears, associated tendon/ neurovascular injury	Not reported	Not reported	Not reported	Not reported	Outcome data complete
Saetta *et al.* ^18^	1992	Unstable MCPJ but specifics not mentioned		Not reported	Not reported	Not reported	Not reported	Outcome data incomplete and author not contactable
Sollerman *et al.* ^15^	1991	Clinical and radiographic assessment but specifics not mentioned		Not reported	Not reported	Not reported	Not reported	Outcome data incomplete and author not contactable

UCL, ulnar collateral ligament; MCPJ, metacarpophalangeal joint.

The study by Sollerman *et al.* ^15^ compared a functional splint with plaster cast treatment in patients with complete UCL ruptures; patients were managed both surgically and non surgically. The authors reported no difference in MCPJ range of movement (ROM), grip strength and sick leave taken; however, the data provided were insufficient for any further analysis, such as a forest plot.

The RCT by Rocchi *et al.* compared the outcomes of operated patients treated with either a traditional standard thumb spica which immobilized the MCPJ or a new modified thumb spica which allowed early MCP motion ^14^. At 12 months the new spica group had increased MCPJ ROM (standardized mean difference (SMD), −3.69; 95% confidence interval (CI), −2.46–−4.92, P<0.0001), a better Dreiser index (SMD, 1.65; 95%CI, 0.81–2.50; P=0.0001) and reduced pain VAS (SMD, 1.53; 95% CI, 0.70–2.35; P=0.0003). There was no statistically significant difference between groups in tip pinch strength at any time point. The RCT by Crowley *et al.* compared outcomes between patients treated with early active mobilization or plaster immobilization after being treated surgically with Mitek anchor repair ^13^. The outcome data was not provided, meaning that any further analysis was not possible.

The retrospective comparative case series by Saetta *et al.* demonstrated a higher chance of an excellent functional result with suture repair versus steel wire, but this was not statistically significant (risk ratio, 1.19; 95% CI, 0.82–1.71); the other outcome data was incomplete and thus precluded further analysis. The retrospective case series by Lane demonstrated no statistically significant difference in the chances of a full versus partial recovery in ROM of the MCPJ, of a full versus partial recovery in strength and of a full versus partial functional recovery ^17^. The study by Katolik *et al.* did not provide adequate data with which to conduct any further analysis ^16^.

### Adverse events

Rocchi *et al* ^14^ *.* demonstrated no statistically significant difference in complication rate between treatment with the standard spica and the new spica (risk ratio, 1.5; 95% CI, 0.29–7.73); the complications consisted of three cases of temporary dysaesthesia and two cases of inflammatory scars. The complication rate was identical in both the early active mobilization and plaster cast groups in the study by Crowley *et al* ^13^ *.* (Risk ratio: 1.0, 95% CI: 0.32, 3.10); all six complications in this study were that of scar tethering, with all resolving with ultrasound therapy and massage. The studies by Saetta *et al.* ^17^ ** and Sollerman *et al.* ^18^ ** did not make any mention of specific complications. Lane ^17^ demonstrated no statistically significant difference in the complication rate between the older method of pull out suture plus K-wire fixation and the new method of suture repair (risk ratio, 3.57; 95% CI, 0.25–50.15); there was one complication with the traditional method (broken pull-out suture at 2 weeks) and one with the new method (re-rupture at 9 months) The study by Katolik *et al* ^16^ *.* demonstrated a higher complication rate with pull-out suture versus bone anchor repair, but this was not statistically significant (risk ratio, 4.00; 95% CI, 0.92–17.30); all the ten complications were soft-tissue-related (five were persistent wound erythema consistent with wound infection and five were paraesthesiae, which resolved over time).

### Risk of bias

**Figure 2.**
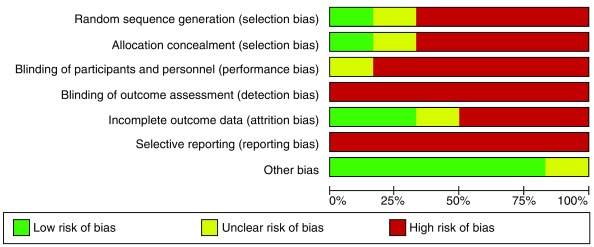
Risk of bias graph.

**Figure 3.**
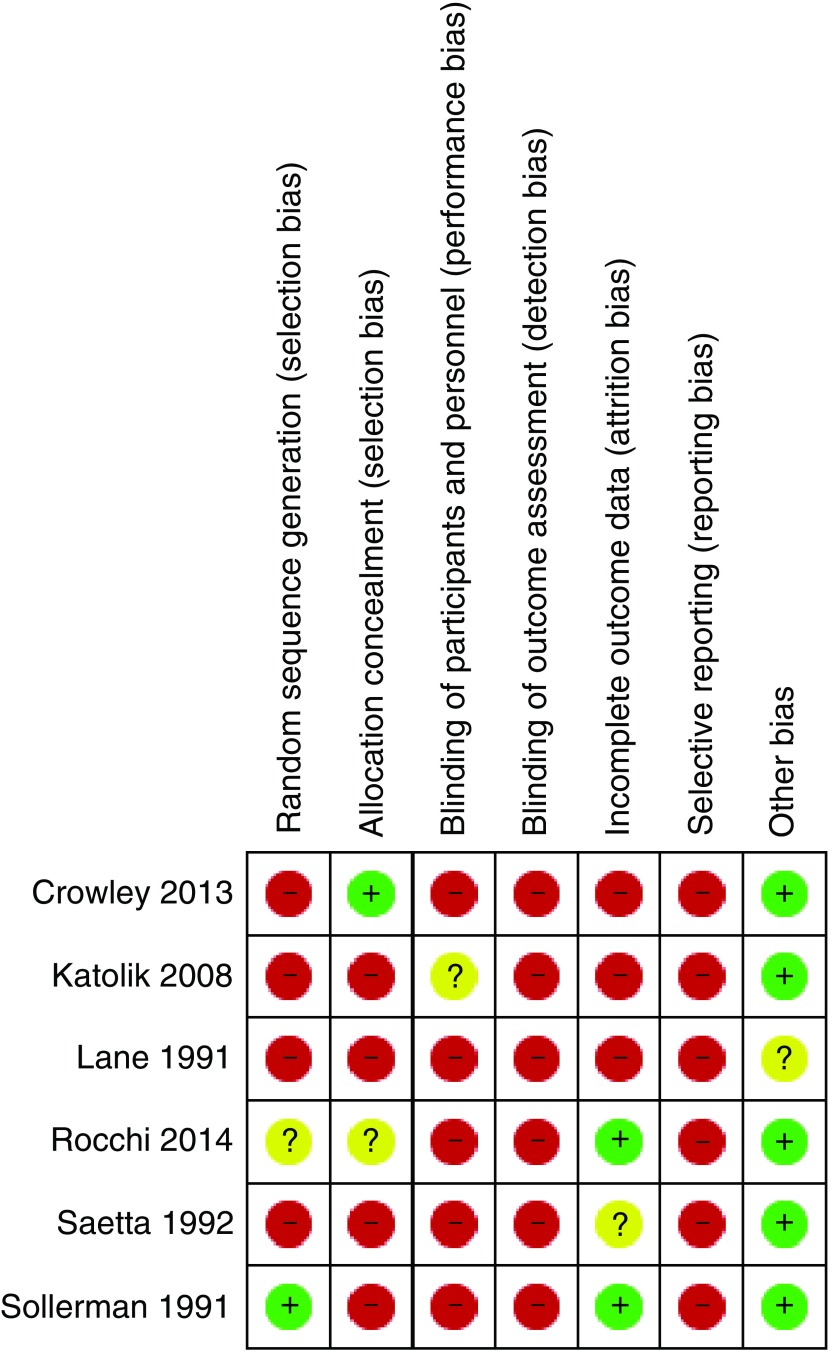
Risk of bias summary.

All criteria were judged as low, high or unclear risk of bias. Overall, all studies were deemed to be at a high risk of bias, particularly in terms of blinding of outcome assessment and selecting reporting. Full risk of bias assessment is available in Figure 2 and Figure 3.

### Meta analysis

As a result of the degree of heterogeneity in terms of study interventions and the incomplete outcome data, it was determined that a meta-analysis of the outcomes was not possible. We carried out a meta-analysis of the complications of pull-out suture versus bone anchor, as two studies had compared these different surgical techniques ^16, 17^. The complication rate of pull out suture fixation was higher than that of bone anchor repair (risk ratio, 3.92; 95% CI, 1.07–14.32; P=0.04). Although suggesting a higher rate of complication, this should be interpreted with caution due to the high risk of bias in the included studes, reducing the reliability of the data and subsequent meta-analysis.

## Discussion

The key finding of this systematic review is that is that no study exists comparing non-operative to surgical intervention in the treatment of complete ruptures of the UCL of the thumb. The only studies which have compared interventions are at high risk of bias, particularly in the areas of blinding of outcome assessment and selective outcome reporting. There is weak evidence to suggest that early mobilisation of the thumb MCPJ may be beneficial following surgical repair. There is weak evidence that the pull out suture fixation has a higher rate of adverse events when compared to bone anchor repair.

A systematic review by Samora *et al.* summarised the outcomes after both non-operative and operative treatment of complete UCL ruptures ^19^. They found that the vast majority of the evidence base was low quality retrospective case series and that only a small minority of patients were treated non-operatively. It was also shown that there was no significant difference in outcome between repair of acute injury and reconstruction after chronic injury.

Landsman *et al.* demonstrated generally good results when managing complete ruptures with splintage with only 15% failing this regime non operative treatment ^4^; notably, 30% of the patients in this series had displaced fractures and all patients had more than 30° laxity in 30° of MCPJ flexion. A case series reported by Pichora *et al.* also demonstrated generally satisfactory functional results with functional bracing, even in the 5 patients who were judged to have sustained true Stener lesions ^20^; notably, the three patients who failed functional bracing could not be predicted by the initial clinical tests. Case series purely relating to avulsion fractures of the UCL have shown contrasting results. For example Kuz *et al.* ^21^ ** demonstrated satisfactory outcomes in all patients but a non union rate of 25%, this contrasts with the results of Dinowitz *et al.* ^22^, ** which demonstrated poor functional results in patients treated non-operatively for minimally displaced fractures.

There is a widely varying rate of Stener lesions in the literature, it being as low as 12% in the series by Pichora *et al.* ^20^ and as high as 70% in other series ^21^. The reasons underlying the variability in the rate of the Stener lesion are likely multiple and complex. One aspect of this conundrum appears to be the clear problems with the reliability and accuracy of the radiological diagnosis of the Stener lesion, particularly relating to MRI ^3^. Although there are some high quality studies describing the reliability of ultrasound, there are no high quality studies relating to MRI ^3^. Mahajan *et al.* demonstrated excellent agreement between radiologists in determining whether the UCL had completely ruptured; however, the presence or absence of a Stener lesion was not assessed radiologically ^6^. Milner *et al.* have recently argued that any displacement of greater than 3 mm (grade 3 by their system) should be treated operatively, owing to the observed high chance that these patients will fail with non operative treatment ^9^.

The recent study by Stoop *et al.* assessed 383 UCL injuries treated at three different hospitals in a singe American city ^11^. In total, 30% of cases were avulsion fractures and 11% of cases were investigated with an MRI scan. Certain patient characteristics were associated with a higher rate of operative intervention, for example greater age and more displaced fractures. However some factors which were unrelated to patient characteristics were predictive of operative intervention, such as having an MRI and being treated by certain surgeons. It was felt that because the preoperative diagnosis of a Stener lesion has limited reliability and accuracy, the rates of surgery may vary based on surgeon beliefs, preferences and values.They also stated that ”some surgeons believe some non-Stener injuries benefit from operative treatment” ^11^.

This review has demonstrated that all six studies of an intervention with a comparator in UCL treatment are at high risk of bias. The blinding of participants would clearly not be possible in a trial of surgery versus non operative treatment; however, it is a recurrent theme that outcomes were assessed by non-blinded assessors (often the treating surgeon), which significantly increases the chance that detection bias will influence patient outcomes. None of the RCTs published a trial protocol with a specified primary outcome, while only the study by Rocchi *et al.* ^14^ ** used validated patient-reported outcome measures (Dreiser index and VAS). There was also a failure to adequately report all outcomes, with only one study reporting adequate data for all outcomes to allow further analysis. None of the three RCTs included a power calculation. While the retrospective nature of the comparative case series introduces several potential sources of bias which may have influenced these results.

## Conclusions

There is no prospective randomised or observational evidence to support operative intervention compared to non-operative treatment for acute complete ruptures of the ulnar collateral ligament of the thumb. There is weak evidence to suggest that early mobilisation may be beneficial following surgical repair. Further research is necessary in order to better define which patients benefit from which specific interventions.

## Data availability

All data underlying the results are available as part of the article and no additional source data are required.

## Supplementary material

**Supplementary File 1. PRISMA checklist.**

**Supplementary File 2. Full search histories.**

**Supplementary File 3. Forest plot of Crowley *et al.*^13^ risk ratio of adverse events.**

**Supplementary File 4. Forest plot of Katolik *et al.*^16^ risk ratio of adverse events.**

**Supplementary File 5. Forest plot of Lane *et al.*^17^ risk ratio of adverse events.**

**Supplementary File 6. Forest plot of Lane *et al.*^17^ risk ratio of full versus partial ROM.**

**Supplementary File 7. Forest plot of Lane *et al.*^17^ risk ration of overall outcome full versus partial.**

**Supplementary File 8. Forest plot of Lane *et al.*^17^ strength recovery full versus partial risk ratio.**

**Supplementary File 9. Forest plot of pull out suture versus anchor complication risk ratio.**

**Supplementary File 10. Forest plot of Rocchi *et al.*^14^ risk ratio of adverse events.**

**Supplementary File 11. Forest plot of Rocchi *et al.*^14^ Dreiser index.**

**Supplementary File 12. Forest plot of Rocchi *et al.*^14^ Range of movement.**

**Supplementary File 13. Forest plot of Rocchi *et al.*^14^ VAS.**

**Supplementary File 14. Forest plot of Rocchi *et al.*^14^ pinch strength.**

**Supplementary File 15. Forest plot of Saetta *et al.*^18^ risk ratio of adverse events.**

**Supplementary File 16. RevMan 5 file containing the full data extracted from the studies.**
