# Supplementary material for: Therapeutic interventions for acute complete ruptures of the ulnar collateral ligament of the thumb: a systematic review
Source: F1000Res. 2018 Jun 8;7:714. [Version 1] doi: 10.12688/f1000research.15065.1 (PMC6051197; doi:10.12688/f1000research.15065.1)
Supplement: Supplementary file 2 [file f1000research-7-16402-s0001.tgz › b3d3210b-d58b-4090-a442-07bcbe455ad8.docx]

**MEDLINE**: (31/01/18)

| **Searches** | **Results** | **Type** | **Actions** | **Annotations** |  |  |
| --- | --- | --- | --- | --- | --- | --- |
|  | | | | | | |
|  | 1 | Collateral Ligament, Ulnar/ | 47 | Advanced | [Display Results](http://ovidsp.tx.ovid.com/sp-3.27.2b/ovidweb.cgi?&S=PECEFPLFJJDDOIBPNCFKLHFBKMGGAA00&SELECT=S.sh%7c&R=1&Process+Action=display)  [More](http://ovidsp.tx.ovid.com/sp-3.27.2b/ovidweb.cgi?&S=PECEFPLFJJDDOIBPNCFKLHFBKMGGAA00&SELECT=S.sh%7c&Expand=1&Main+Search+Page=Main+Search+Page) | [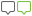](http://ovidsp.tx.ovid.com/sp-3.27.2b/ovidweb.cgi?&S=PECEFPLFJJDDOIBPNCFKLHFBKMGGAA00&R=1&Search+Annotations+Options=SA) |
|  | 2 | (ulnar adj3 collateral ligament*).ti,ab. | 888 | Advanced | [Display Results](http://ovidsp.tx.ovid.com/sp-3.27.2b/ovidweb.cgi?&S=PECEFPLFJJDDOIBPNCFKLHFBKMGGAA00&SELECT=S.sh%7c&R=2&Process+Action=display)  [More](http://ovidsp.tx.ovid.com/sp-3.27.2b/ovidweb.cgi?&S=PECEFPLFJJDDOIBPNCFKLHFBKMGGAA00&SELECT=S.sh%7c&Expand=1&Main+Search+Page=Main+Search+Page) | [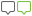](http://ovidsp.tx.ovid.com/sp-3.27.2b/ovidweb.cgi?&S=PECEFPLFJJDDOIBPNCFKLHFBKMGGAA00&R=2&Search+Annotations+Options=SA) |
|  | 3 | ucl.ti,ab. | 992 | Advanced | [Display Results](http://ovidsp.tx.ovid.com/sp-3.27.2b/ovidweb.cgi?&S=PECEFPLFJJDDOIBPNCFKLHFBKMGGAA00&SELECT=S.sh%7c&R=3&Process+Action=display)  [More](http://ovidsp.tx.ovid.com/sp-3.27.2b/ovidweb.cgi?&S=PECEFPLFJJDDOIBPNCFKLHFBKMGGAA00&SELECT=S.sh%7c&Expand=1&Main+Search+Page=Main+Search+Page) | [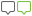](http://ovidsp.tx.ovid.com/sp-3.27.2b/ovidweb.cgi?&S=PECEFPLFJJDDOIBPNCFKLHFBKMGGAA00&R=3&Search+Annotations+Options=SA) |
|  | 4 | Metacarpophalangeal Joint/ | 2897 | Advanced | [Display Results](http://ovidsp.tx.ovid.com/sp-3.27.2b/ovidweb.cgi?&S=PECEFPLFJJDDOIBPNCFKLHFBKMGGAA00&SELECT=S.sh%7c&R=4&Process+Action=display)  [More](http://ovidsp.tx.ovid.com/sp-3.27.2b/ovidweb.cgi?&S=PECEFPLFJJDDOIBPNCFKLHFBKMGGAA00&SELECT=S.sh%7c&Expand=1&Main+Search+Page=Main+Search+Page) | [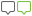](http://ovidsp.tx.ovid.com/sp-3.27.2b/ovidweb.cgi?&S=PECEFPLFJJDDOIBPNCFKLHFBKMGGAA00&R=4&Search+Annotations+Options=SA) |
|  | 5 | Collateral Ligaments/ | 1435 | Advanced | [Display Results](http://ovidsp.tx.ovid.com/sp-3.27.2b/ovidweb.cgi?&S=PECEFPLFJJDDOIBPNCFKLHFBKMGGAA00&SELECT=S.sh%7c&R=5&Process+Action=display)  [More](http://ovidsp.tx.ovid.com/sp-3.27.2b/ovidweb.cgi?&S=PECEFPLFJJDDOIBPNCFKLHFBKMGGAA00&SELECT=S.sh%7c&Expand=1&Main+Search+Page=Main+Search+Page) | [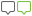](http://ovidsp.tx.ovid.com/sp-3.27.2b/ovidweb.cgi?&S=PECEFPLFJJDDOIBPNCFKLHFBKMGGAA00&R=5&Search+Annotations+Options=SA) |
|  | 6 | mcp.ti,ab. | 19655 | Advanced | [Display Results](http://ovidsp.tx.ovid.com/sp-3.27.2b/ovidweb.cgi?&S=PECEFPLFJJDDOIBPNCFKLHFBKMGGAA00&SELECT=S.sh%7c&R=6&Process+Action=display)  [More](http://ovidsp.tx.ovid.com/sp-3.27.2b/ovidweb.cgi?&S=PECEFPLFJJDDOIBPNCFKLHFBKMGGAA00&SELECT=S.sh%7c&Expand=1&Main+Search+Page=Main+Search+Page) | [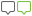](http://ovidsp.tx.ovid.com/sp-3.27.2b/ovidweb.cgi?&S=PECEFPLFJJDDOIBPNCFKLHFBKMGGAA00&R=6&Search+Annotations+Options=SA) |
|  | 7 | (metacarpophalan* adj3 joint*).ti,ab. | 3456 | Advanced | [Display Results](http://ovidsp.tx.ovid.com/sp-3.27.2b/ovidweb.cgi?&S=PECEFPLFJJDDOIBPNCFKLHFBKMGGAA00&SELECT=S.sh%7c&R=7&Process+Action=display)  [More](http://ovidsp.tx.ovid.com/sp-3.27.2b/ovidweb.cgi?&S=PECEFPLFJJDDOIBPNCFKLHFBKMGGAA00&SELECT=S.sh%7c&Expand=1&Main+Search+Page=Main+Search+Page) | [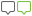](http://ovidsp.tx.ovid.com/sp-3.27.2b/ovidweb.cgi?&S=PECEFPLFJJDDOIBPNCFKLHFBKMGGAA00&R=7&Search+Annotations+Options=SA) |
|  | 8 | (metacarpal phalan* adj3 joint*).ti,ab. | 100 | Advanced | [Display Results](http://ovidsp.tx.ovid.com/sp-3.27.2b/ovidweb.cgi?&S=PECEFPLFJJDDOIBPNCFKLHFBKMGGAA00&SELECT=S.sh%7c&R=8&Process+Action=display)  [More](http://ovidsp.tx.ovid.com/sp-3.27.2b/ovidweb.cgi?&S=PECEFPLFJJDDOIBPNCFKLHFBKMGGAA00&SELECT=S.sh%7c&Expand=1&Main+Search+Page=Main+Search+Page) | [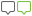](http://ovidsp.tx.ovid.com/sp-3.27.2b/ovidweb.cgi?&S=PECEFPLFJJDDOIBPNCFKLHFBKMGGAA00&R=8&Search+Annotations+Options=SA) |
|  | 9 | Thumb/ | 8425 | Advanced | [Display Results](http://ovidsp.tx.ovid.com/sp-3.27.2b/ovidweb.cgi?&S=PECEFPLFJJDDOIBPNCFKLHFBKMGGAA00&SELECT=S.sh%7c&R=9&Process+Action=display)  [More](http://ovidsp.tx.ovid.com/sp-3.27.2b/ovidweb.cgi?&S=PECEFPLFJJDDOIBPNCFKLHFBKMGGAA00&SELECT=S.sh%7c&Expand=1&Main+Search+Page=Main+Search+Page) | [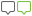](http://ovidsp.tx.ovid.com/sp-3.27.2b/ovidweb.cgi?&S=PECEFPLFJJDDOIBPNCFKLHFBKMGGAA00&R=9&Search+Annotations+Options=SA) |
|  | 10 | thumb*.ti,ab. | 15337 | Advanced | [Display Results](http://ovidsp.tx.ovid.com/sp-3.27.2b/ovidweb.cgi?&S=PECEFPLFJJDDOIBPNCFKLHFBKMGGAA00&SELECT=S.sh%7c&R=10&Process+Action=display)  [More](http://ovidsp.tx.ovid.com/sp-3.27.2b/ovidweb.cgi?&S=PECEFPLFJJDDOIBPNCFKLHFBKMGGAA00&SELECT=S.sh%7c&Expand=1&Main+Search+Page=Main+Search+Page) | [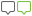](http://ovidsp.tx.ovid.com/sp-3.27.2b/ovidweb.cgi?&S=PECEFPLFJJDDOIBPNCFKLHFBKMGGAA00&R=10&Search+Annotations+Options=SA) |
|  | 11 | pollex.ti,ab. | 59 | Advanced | [Display Results](http://ovidsp.tx.ovid.com/sp-3.27.2b/ovidweb.cgi?&S=PECEFPLFJJDDOIBPNCFKLHFBKMGGAA00&SELECT=S.sh%7c&R=11&Process+Action=display)  [More](http://ovidsp.tx.ovid.com/sp-3.27.2b/ovidweb.cgi?&S=PECEFPLFJJDDOIBPNCFKLHFBKMGGAA00&SELECT=S.sh%7c&Expand=1&Main+Search+Page=Main+Search+Page) | [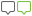](http://ovidsp.tx.ovid.com/sp-3.27.2b/ovidweb.cgi?&S=PECEFPLFJJDDOIBPNCFKLHFBKMGGAA00&R=11&Search+Annotations+Options=SA) |
|  | 12 | (first digit* adj3 hand*).ti,ab. | 7 | Advanced | [Display Results](http://ovidsp.tx.ovid.com/sp-3.27.2b/ovidweb.cgi?&S=PECEFPLFJJDDOIBPNCFKLHFBKMGGAA00&SELECT=S.sh%7c&R=12&Process+Action=display)  [More](http://ovidsp.tx.ovid.com/sp-3.27.2b/ovidweb.cgi?&S=PECEFPLFJJDDOIBPNCFKLHFBKMGGAA00&SELECT=S.sh%7c&Expand=1&Main+Search+Page=Main+Search+Page) | [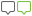](http://ovidsp.tx.ovid.com/sp-3.27.2b/ovidweb.cgi?&S=PECEFPLFJJDDOIBPNCFKLHFBKMGGAA00&R=12&Search+Annotations+Options=SA) |
|  | 13 | (1st digit* adj3 hand*).ti,ab. | 2 | Advanced | [Display Results](http://ovidsp.tx.ovid.com/sp-3.27.2b/ovidweb.cgi?&S=PECEFPLFJJDDOIBPNCFKLHFBKMGGAA00&SELECT=S.sh%7c&R=13&Process+Action=display)  [More](http://ovidsp.tx.ovid.com/sp-3.27.2b/ovidweb.cgi?&S=PECEFPLFJJDDOIBPNCFKLHFBKMGGAA00&SELECT=S.sh%7c&Expand=1&Main+Search+Page=Main+Search+Page) | [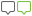](http://ovidsp.tx.ovid.com/sp-3.27.2b/ovidweb.cgi?&S=PECEFPLFJJDDOIBPNCFKLHFBKMGGAA00&R=13&Search+Annotations+Options=SA) |
|  | 14 | (preaxial* adj3 hand*).ti,ab. | 33 | Advanced | [Display Results](http://ovidsp.tx.ovid.com/sp-3.27.2b/ovidweb.cgi?&S=PECEFPLFJJDDOIBPNCFKLHFBKMGGAA00&SELECT=S.sh%7c&R=14&Process+Action=display)  [More](http://ovidsp.tx.ovid.com/sp-3.27.2b/ovidweb.cgi?&S=PECEFPLFJJDDOIBPNCFKLHFBKMGGAA00&SELECT=S.sh%7c&Expand=1&Main+Search+Page=Main+Search+Page) | [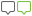](http://ovidsp.tx.ovid.com/sp-3.27.2b/ovidweb.cgi?&S=PECEFPLFJJDDOIBPNCFKLHFBKMGGAA00&R=14&Search+Annotations+Options=SA) |
|  | 15 | (pre axial* adj3 hand*).ti,ab. | 3 | Advanced | [Display Results](http://ovidsp.tx.ovid.com/sp-3.27.2b/ovidweb.cgi?&S=PECEFPLFJJDDOIBPNCFKLHFBKMGGAA00&SELECT=S.sh%7c&R=15&Process+Action=display)  [More](http://ovidsp.tx.ovid.com/sp-3.27.2b/ovidweb.cgi?&S=PECEFPLFJJDDOIBPNCFKLHFBKMGGAA00&SELECT=S.sh%7c&Expand=1&Main+Search+Page=Main+Search+Page) | [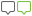](http://ovidsp.tx.ovid.com/sp-3.27.2b/ovidweb.cgi?&S=PECEFPLFJJDDOIBPNCFKLHFBKMGGAA00&R=15&Search+Annotations+Options=SA) |
|  | 16 | 1 or 2 or 3 or 4 or 5 or 6 or 7 or 8 | 26247 | Advanced | [Display Results](http://ovidsp.tx.ovid.com/sp-3.27.2b/ovidweb.cgi?&S=PECEFPLFJJDDOIBPNCFKLHFBKMGGAA00&SELECT=S.sh%7c&R=16&Process+Action=display)  [More](http://ovidsp.tx.ovid.com/sp-3.27.2b/ovidweb.cgi?&S=PECEFPLFJJDDOIBPNCFKLHFBKMGGAA00&SELECT=S.sh%7c&Expand=1&Main+Search+Page=Main+Search+Page) | [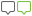](http://ovidsp.tx.ovid.com/sp-3.27.2b/ovidweb.cgi?&S=PECEFPLFJJDDOIBPNCFKLHFBKMGGAA00&R=16&Search+Annotations+Options=SA) |
|  | 17 | 9 or 10 or 11 or 12 or 13 or 14 or 15 | 18188 | Advanced | [Display Results](http://ovidsp.tx.ovid.com/sp-3.27.2b/ovidweb.cgi?&S=PECEFPLFJJDDOIBPNCFKLHFBKMGGAA00&SELECT=S.sh%7c&R=17&Process+Action=display)  [More](http://ovidsp.tx.ovid.com/sp-3.27.2b/ovidweb.cgi?&S=PECEFPLFJJDDOIBPNCFKLHFBKMGGAA00&SELECT=S.sh%7c&Expand=1&Main+Search+Page=Main+Search+Page) | [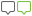](http://ovidsp.tx.ovid.com/sp-3.27.2b/ovidweb.cgi?&S=PECEFPLFJJDDOIBPNCFKLHFBKMGGAA00&R=17&Search+Annotations+Options=SA) |
|  | 18 | 16 and 17 | 1561 | Advanced | [Display Results](http://ovidsp.tx.ovid.com/sp-3.27.2b/ovidweb.cgi?&S=PECEFPLFJJDDOIBPNCFKLHFBKMGGAA00&SELECT=S.sh%7c&R=18&Process+Action=display)  [More](http://ovidsp.tx.ovid.com/sp-3.27.2b/ovidweb.cgi?&S=PECEFPLFJJDDOIBPNCFKLHFBKMGGAA00&SELECT=S.sh%7c&Expand=1&Main+Search+Page=Main+Search+Page) | [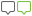](http://ovidsp.tx.ovid.com/sp-3.27.2b/ovidweb.cgi?&S=PECEFPLFJJDDOIBPNCFKLHFBKMGGAA00&R=18&Search+Annotations+Options=SA) |
|  | 19 | gamekeepers thumb*.ti,ab. | 49 | Advanced | [Display Results](http://ovidsp.tx.ovid.com/sp-3.27.2b/ovidweb.cgi?&S=PECEFPLFJJDDOIBPNCFKLHFBKMGGAA00&SELECT=S.sh%7c&R=19&Process+Action=display)  [More](http://ovidsp.tx.ovid.com/sp-3.27.2b/ovidweb.cgi?&S=PECEFPLFJJDDOIBPNCFKLHFBKMGGAA00&SELECT=S.sh%7c&Expand=1&Main+Search+Page=Main+Search+Page) | [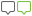](http://ovidsp.tx.ovid.com/sp-3.27.2b/ovidweb.cgi?&S=PECEFPLFJJDDOIBPNCFKLHFBKMGGAA00&R=19&Search+Annotations+Options=SA) |
|  | 20 | skiers thumb*.ti,ab. | 47 | Advanced | [Display Results](http://ovidsp.tx.ovid.com/sp-3.27.2b/ovidweb.cgi?&S=PECEFPLFJJDDOIBPNCFKLHFBKMGGAA00&SELECT=S.sh%7c&R=20&Process+Action=display)  [More](http://ovidsp.tx.ovid.com/sp-3.27.2b/ovidweb.cgi?&S=PECEFPLFJJDDOIBPNCFKLHFBKMGGAA00&SELECT=S.sh%7c&Expand=1&Main+Search+Page=Main+Search+Page) | [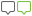](http://ovidsp.tx.ovid.com/sp-3.27.2b/ovidweb.cgi?&S=PECEFPLFJJDDOIBPNCFKLHFBKMGGAA00&R=20&Search+Annotations+Options=SA) |
|  | 21 | 18 or 19 or 20 | 1584 | Advanced | [Display Results](http://ovidsp.tx.ovid.com/sp-3.27.2b/ovidweb.cgi?&S=PECEFPLFJJDDOIBPNCFKLHFBKMGGAA00&SELECT=S.sh%7c&R=21&Process+Action=display)  [More](http://ovidsp.tx.ovid.com/sp-3.27.2b/ovidweb.cgi?&S=PECEFPLFJJDDOIBPNCFKLHFBKMGGAA00&SELECT=S.sh%7c&Expand=1&Main+Search+Page=Main+Search+Page) | [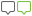](http://ovidsp.tx.ovid.com/sp-3.27.2b/ovidweb.cgi?&S=PECEFPLFJJDDOIBPNCFKLHFBKMGGAA00&R=21&Search+Annotations+Options=SA) |
|  | 22 | randomized controlled trial.pt. | 452080 | Advanced | [Display Results](http://ovidsp.tx.ovid.com/sp-3.27.2b/ovidweb.cgi?&S=PECEFPLFJJDDOIBPNCFKLHFBKMGGAA00&SELECT=S.sh%7c&R=22&Process+Action=display)  [More](http://ovidsp.tx.ovid.com/sp-3.27.2b/ovidweb.cgi?&S=PECEFPLFJJDDOIBPNCFKLHFBKMGGAA00&SELECT=S.sh%7c&Expand=1&Main+Search+Page=Main+Search+Page) | [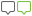](http://ovidsp.tx.ovid.com/sp-3.27.2b/ovidweb.cgi?&S=PECEFPLFJJDDOIBPNCFKLHFBKMGGAA00&R=22&Search+Annotations+Options=SA) |
|  | 23 | controlled clinical trial.pt. | 92108 | Advanced | [Display Results](http://ovidsp.tx.ovid.com/sp-3.27.2b/ovidweb.cgi?&S=PECEFPLFJJDDOIBPNCFKLHFBKMGGAA00&SELECT=S.sh%7c&R=23&Process+Action=display)  [More](http://ovidsp.tx.ovid.com/sp-3.27.2b/ovidweb.cgi?&S=PECEFPLFJJDDOIBPNCFKLHFBKMGGAA00&SELECT=S.sh%7c&Expand=1&Main+Search+Page=Main+Search+Page) | [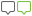](http://ovidsp.tx.ovid.com/sp-3.27.2b/ovidweb.cgi?&S=PECEFPLFJJDDOIBPNCFKLHFBKMGGAA00&R=23&Search+Annotations+Options=SA) |
|  | 24 | randomized.ab. | 400977 | Advanced | [Display Results](http://ovidsp.tx.ovid.com/sp-3.27.2b/ovidweb.cgi?&S=PECEFPLFJJDDOIBPNCFKLHFBKMGGAA00&SELECT=S.sh%7c&R=24&Process+Action=display)  [More](http://ovidsp.tx.ovid.com/sp-3.27.2b/ovidweb.cgi?&S=PECEFPLFJJDDOIBPNCFKLHFBKMGGAA00&SELECT=S.sh%7c&Expand=1&Main+Search+Page=Main+Search+Page) | [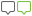](http://ovidsp.tx.ovid.com/sp-3.27.2b/ovidweb.cgi?&S=PECEFPLFJJDDOIBPNCFKLHFBKMGGAA00&R=24&Search+Annotations+Options=SA) |
|  | 25 | placebo.ab. | 185887 | Advanced | [Display Results](http://ovidsp.tx.ovid.com/sp-3.27.2b/ovidweb.cgi?&S=PECEFPLFJJDDOIBPNCFKLHFBKMGGAA00&SELECT=S.sh%7c&R=25&Process+Action=display)  [More](http://ovidsp.tx.ovid.com/sp-3.27.2b/ovidweb.cgi?&S=PECEFPLFJJDDOIBPNCFKLHFBKMGGAA00&SELECT=S.sh%7c&Expand=1&Main+Search+Page=Main+Search+Page) | [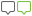](http://ovidsp.tx.ovid.com/sp-3.27.2b/ovidweb.cgi?&S=PECEFPLFJJDDOIBPNCFKLHFBKMGGAA00&R=25&Search+Annotations+Options=SA) |
|  | 26 | drug therapy.fs. | 1987533 | Advanced | [Display Results](http://ovidsp.tx.ovid.com/sp-3.27.2b/ovidweb.cgi?&S=PECEFPLFJJDDOIBPNCFKLHFBKMGGAA00&SELECT=S.sh%7c&R=26&Process+Action=display)  [More](http://ovidsp.tx.ovid.com/sp-3.27.2b/ovidweb.cgi?&S=PECEFPLFJJDDOIBPNCFKLHFBKMGGAA00&SELECT=S.sh%7c&Expand=1&Main+Search+Page=Main+Search+Page) | [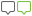](http://ovidsp.tx.ovid.com/sp-3.27.2b/ovidweb.cgi?&S=PECEFPLFJJDDOIBPNCFKLHFBKMGGAA00&R=26&Search+Annotations+Options=SA) |
|  | 27 | randomly.ab. | 283901 | Advanced | [Display Results](http://ovidsp.tx.ovid.com/sp-3.27.2b/ovidweb.cgi?&S=PECEFPLFJJDDOIBPNCFKLHFBKMGGAA00&SELECT=S.sh%7c&R=27&Process+Action=display)  [More](http://ovidsp.tx.ovid.com/sp-3.27.2b/ovidweb.cgi?&S=PECEFPLFJJDDOIBPNCFKLHFBKMGGAA00&SELECT=S.sh%7c&Expand=1&Main+Search+Page=Main+Search+Page) | [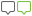](http://ovidsp.tx.ovid.com/sp-3.27.2b/ovidweb.cgi?&S=PECEFPLFJJDDOIBPNCFKLHFBKMGGAA00&R=27&Search+Annotations+Options=SA) |
|  | 28 | trial.ab. | 416178 | Advanced | [Display Results](http://ovidsp.tx.ovid.com/sp-3.27.2b/ovidweb.cgi?&S=PECEFPLFJJDDOIBPNCFKLHFBKMGGAA00&SELECT=S.sh%7c&R=28&Process+Action=display)  [More](http://ovidsp.tx.ovid.com/sp-3.27.2b/ovidweb.cgi?&S=PECEFPLFJJDDOIBPNCFKLHFBKMGGAA00&SELECT=S.sh%7c&Expand=1&Main+Search+Page=Main+Search+Page) | [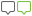](http://ovidsp.tx.ovid.com/sp-3.27.2b/ovidweb.cgi?&S=PECEFPLFJJDDOIBPNCFKLHFBKMGGAA00&R=28&Search+Annotations+Options=SA) |
|  | 29 | groups.ab. | 1756874 | Advanced | [Display Results](http://ovidsp.tx.ovid.com/sp-3.27.2b/ovidweb.cgi?&S=PECEFPLFJJDDOIBPNCFKLHFBKMGGAA00&SELECT=S.sh%7c&R=29&Process+Action=display)  [More](http://ovidsp.tx.ovid.com/sp-3.27.2b/ovidweb.cgi?&S=PECEFPLFJJDDOIBPNCFKLHFBKMGGAA00&SELECT=S.sh%7c&Expand=1&Main+Search+Page=Main+Search+Page) | [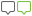](http://ovidsp.tx.ovid.com/sp-3.27.2b/ovidweb.cgi?&S=PECEFPLFJJDDOIBPNCFKLHFBKMGGAA00&R=29&Search+Annotations+Options=SA) |
|  | 30 | 22 or 23 or 24 or 25 or 26 or 27 or 28 or 29 | 4126497 | Advanced | [Display Results](http://ovidsp.tx.ovid.com/sp-3.27.2b/ovidweb.cgi?&S=PECEFPLFJJDDOIBPNCFKLHFBKMGGAA00&SELECT=S.sh%7c&R=30&Process+Action=display)  [More](http://ovidsp.tx.ovid.com/sp-3.27.2b/ovidweb.cgi?&S=PECEFPLFJJDDOIBPNCFKLHFBKMGGAA00&SELECT=S.sh%7c&Expand=1&Main+Search+Page=Main+Search+Page) | [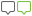](http://ovidsp.tx.ovid.com/sp-3.27.2b/ovidweb.cgi?&S=PECEFPLFJJDDOIBPNCFKLHFBKMGGAA00&R=30&Search+Annotations+Options=SA) |
|  | 31 | exp animals/ not humans.sh. | 4418500 | Advanced | [Display Results](http://ovidsp.tx.ovid.com/sp-3.27.2b/ovidweb.cgi?&S=PECEFPLFJJDDOIBPNCFKLHFBKMGGAA00&SELECT=S.sh%7c&R=31&Process+Action=display)  [More](http://ovidsp.tx.ovid.com/sp-3.27.2b/ovidweb.cgi?&S=PECEFPLFJJDDOIBPNCFKLHFBKMGGAA00&SELECT=S.sh%7c&Expand=1&Main+Search+Page=Main+Search+Page) | [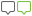](http://ovidsp.tx.ovid.com/sp-3.27.2b/ovidweb.cgi?&S=PECEFPLFJJDDOIBPNCFKLHFBKMGGAA00&R=31&Search+Annotations+Options=SA) |
|  | 32 | 30 not 31 | 3564448 | Advanced | [Display Results](http://ovidsp.tx.ovid.com/sp-3.27.2b/ovidweb.cgi?&S=PECEFPLFJJDDOIBPNCFKLHFBKMGGAA00&SELECT=S.sh%7c&R=32&Process+Action=display)  [More](http://ovidsp.tx.ovid.com/sp-3.27.2b/ovidweb.cgi?&S=PECEFPLFJJDDOIBPNCFKLHFBKMGGAA00&SELECT=S.sh%7c&Expand=1&Main+Search+Page=Main+Search+Page) | [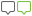](http://ovidsp.tx.ovid.com/sp-3.27.2b/ovidweb.cgi?&S=PECEFPLFJJDDOIBPNCFKLHFBKMGGAA00&R=32&Search+Annotations+Options=SA) |
|  | 33 | 21 and 32 | 117 | Advanced | [Display Results](http://ovidsp.tx.ovid.com/sp-3.27.2b/ovidweb.cgi?&S=PECEFPLFJJDDOIBPNCFKLHFBKMGGAA00&SELECT=S.sh%7c&R=33&Process+Action=display)  [More](http://ovidsp.tx.ovid.com/sp-3.27.2b/ovidweb.cgi?&S=PECEFPLFJJDDOIBPNCFKLHFBKMGGAA00&SELECT=S.sh%7c&Expand=1&Main+Search+Page=Main+Search+Page) | [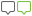](http://ovidsp.tx.ovid.com/sp-3.27.2b/ovidweb.cgi?&S=PECEFPLFJJDDOIBPNCFKLHFBKMGGAA00&R=33&Search+Annotations+Options=SA) |
|  | | |  |  |  |  |

Combine with:

|  |  |
| --- | --- |

**EMBASE:** (31/01/18)

| **Searches** | **Results** | **Type** | **Actions** | **Annotations** |  |  |
| --- | --- | --- | --- | --- | --- | --- |
|  | | | | | | |
|  | 1 | "ulnar collateral ligament (thumb)"/ | 11 | Advanced | [Display Results](http://ovidsp.tx.ovid.com/sp-3.27.2b/ovidweb.cgi?&S=FOPEFPHDIGDDOIIENCFKDFMCGGNNAA00&SELECT=S.sh%7c&R=1&Process+Action=display)  [More](http://ovidsp.tx.ovid.com/sp-3.27.2b/ovidweb.cgi?&S=FOPEFPHDIGDDOIIENCFKDFMCGGNNAA00&SELECT=S.sh%7c&Expand=1&Main+Search+Page=Main+Search+Page) | [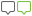](http://ovidsp.tx.ovid.com/sp-3.27.2b/ovidweb.cgi?&S=FOPEFPHDIGDDOIIENCFKDFMCGGNNAA00&R=1&Search+Annotations+Options=SA) |
|  | 2 | (ulnar adj3 collateral ligament*).ti,ab. | 982 | Advanced | [Display Results](http://ovidsp.tx.ovid.com/sp-3.27.2b/ovidweb.cgi?&S=FOPEFPHDIGDDOIIENCFKDFMCGGNNAA00&SELECT=S.sh%7c&R=2&Process+Action=display)  [More](http://ovidsp.tx.ovid.com/sp-3.27.2b/ovidweb.cgi?&S=FOPEFPHDIGDDOIIENCFKDFMCGGNNAA00&SELECT=S.sh%7c&Expand=1&Main+Search+Page=Main+Search+Page) | [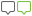](http://ovidsp.tx.ovid.com/sp-3.27.2b/ovidweb.cgi?&S=FOPEFPHDIGDDOIIENCFKDFMCGGNNAA00&R=2&Search+Annotations+Options=SA) |
|  | 3 | ucl.ti,ab. | 1332 | Advanced | [Display Results](http://ovidsp.tx.ovid.com/sp-3.27.2b/ovidweb.cgi?&S=FOPEFPHDIGDDOIIENCFKDFMCGGNNAA00&SELECT=S.sh%7c&R=3&Process+Action=display)  [More](http://ovidsp.tx.ovid.com/sp-3.27.2b/ovidweb.cgi?&S=FOPEFPHDIGDDOIIENCFKDFMCGGNNAA00&SELECT=S.sh%7c&Expand=1&Main+Search+Page=Main+Search+Page) | [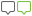](http://ovidsp.tx.ovid.com/sp-3.27.2b/ovidweb.cgi?&S=FOPEFPHDIGDDOIIENCFKDFMCGGNNAA00&R=3&Search+Annotations+Options=SA) |
|  | 4 | *metacarpophalangeal joint/ | 1554 | Advanced | [Display Results](http://ovidsp.tx.ovid.com/sp-3.27.2b/ovidweb.cgi?&S=FOPEFPHDIGDDOIIENCFKDFMCGGNNAA00&SELECT=S.sh%7c&R=4&Process+Action=display)  [More](http://ovidsp.tx.ovid.com/sp-3.27.2b/ovidweb.cgi?&S=FOPEFPHDIGDDOIIENCFKDFMCGGNNAA00&SELECT=S.sh%7c&Expand=1&Main+Search+Page=Main+Search+Page) | [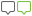](http://ovidsp.tx.ovid.com/sp-3.27.2b/ovidweb.cgi?&S=FOPEFPHDIGDDOIIENCFKDFMCGGNNAA00&R=4&Search+Annotations+Options=SA) |
|  | 5 | metacarpophalangeal joint/ | 5583 | Advanced | [Display Results](http://ovidsp.tx.ovid.com/sp-3.27.2b/ovidweb.cgi?&S=FOPEFPHDIGDDOIIENCFKDFMCGGNNAA00&SELECT=S.sh%7c&R=5&Process+Action=display)  [More](http://ovidsp.tx.ovid.com/sp-3.27.2b/ovidweb.cgi?&S=FOPEFPHDIGDDOIIENCFKDFMCGGNNAA00&SELECT=S.sh%7c&Expand=1&Main+Search+Page=Main+Search+Page) | [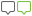](http://ovidsp.tx.ovid.com/sp-3.27.2b/ovidweb.cgi?&S=FOPEFPHDIGDDOIIENCFKDFMCGGNNAA00&R=5&Search+Annotations+Options=SA) |
|  | 6 | (metacarpophalan* adj3 joint*).ti,ab. | 4169 | Advanced | [Display Results](http://ovidsp.tx.ovid.com/sp-3.27.2b/ovidweb.cgi?&S=FOPEFPHDIGDDOIIENCFKDFMCGGNNAA00&SELECT=S.sh%7c&R=6&Process+Action=display)  [More](http://ovidsp.tx.ovid.com/sp-3.27.2b/ovidweb.cgi?&S=FOPEFPHDIGDDOIIENCFKDFMCGGNNAA00&SELECT=S.sh%7c&Expand=1&Main+Search+Page=Main+Search+Page) | [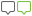](http://ovidsp.tx.ovid.com/sp-3.27.2b/ovidweb.cgi?&S=FOPEFPHDIGDDOIIENCFKDFMCGGNNAA00&R=6&Search+Annotations+Options=SA) |
|  | 7 | (metacarpal phalan* adj3 joint*).ti,ab. | 138 | Advanced | [Display Results](http://ovidsp.tx.ovid.com/sp-3.27.2b/ovidweb.cgi?&S=FOPEFPHDIGDDOIIENCFKDFMCGGNNAA00&SELECT=S.sh%7c&R=7&Process+Action=display)  [More](http://ovidsp.tx.ovid.com/sp-3.27.2b/ovidweb.cgi?&S=FOPEFPHDIGDDOIIENCFKDFMCGGNNAA00&SELECT=S.sh%7c&Expand=1&Main+Search+Page=Main+Search+Page) | [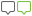](http://ovidsp.tx.ovid.com/sp-3.27.2b/ovidweb.cgi?&S=FOPEFPHDIGDDOIIENCFKDFMCGGNNAA00&R=7&Search+Annotations+Options=SA) |
|  | 8 | exp *collateral ligament/ | 279 | Advanced | [Display Results](http://ovidsp.tx.ovid.com/sp-3.27.2b/ovidweb.cgi?&S=FOPEFPHDIGDDOIIENCFKDFMCGGNNAA00&SELECT=S.sh%7c&R=8&Process+Action=display)  [More](http://ovidsp.tx.ovid.com/sp-3.27.2b/ovidweb.cgi?&S=FOPEFPHDIGDDOIIENCFKDFMCGGNNAA00&SELECT=S.sh%7c&Expand=1&Main+Search+Page=Main+Search+Page) | [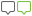](http://ovidsp.tx.ovid.com/sp-3.27.2b/ovidweb.cgi?&S=FOPEFPHDIGDDOIIENCFKDFMCGGNNAA00&R=8&Search+Annotations+Options=SA) |
|  | 9 | exp collateral ligament/ | 885 | Advanced | [Display Results](http://ovidsp.tx.ovid.com/sp-3.27.2b/ovidweb.cgi?&S=FOPEFPHDIGDDOIIENCFKDFMCGGNNAA00&SELECT=S.sh%7c&R=9&Process+Action=display)  [More](http://ovidsp.tx.ovid.com/sp-3.27.2b/ovidweb.cgi?&S=FOPEFPHDIGDDOIIENCFKDFMCGGNNAA00&SELECT=S.sh%7c&Expand=1&Main+Search+Page=Main+Search+Page) | [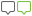](http://ovidsp.tx.ovid.com/sp-3.27.2b/ovidweb.cgi?&S=FOPEFPHDIGDDOIIENCFKDFMCGGNNAA00&R=9&Search+Annotations+Options=SA) |
|  | 10 | mcp.ti,ab. | 29908 | Advanced | [Display Results](http://ovidsp.tx.ovid.com/sp-3.27.2b/ovidweb.cgi?&S=FOPEFPHDIGDDOIIENCFKDFMCGGNNAA00&SELECT=S.sh%7c&R=10&Process+Action=display)  [More](http://ovidsp.tx.ovid.com/sp-3.27.2b/ovidweb.cgi?&S=FOPEFPHDIGDDOIIENCFKDFMCGGNNAA00&SELECT=S.sh%7c&Expand=1&Main+Search+Page=Main+Search+Page) | [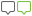](http://ovidsp.tx.ovid.com/sp-3.27.2b/ovidweb.cgi?&S=FOPEFPHDIGDDOIIENCFKDFMCGGNNAA00&R=10&Search+Annotations+Options=SA) |
|  | 11 | *thumb/ | 3199 | Advanced | [Display Results](http://ovidsp.tx.ovid.com/sp-3.27.2b/ovidweb.cgi?&S=FOPEFPHDIGDDOIIENCFKDFMCGGNNAA00&SELECT=S.sh%7c&R=11&Process+Action=display)  [More](http://ovidsp.tx.ovid.com/sp-3.27.2b/ovidweb.cgi?&S=FOPEFPHDIGDDOIIENCFKDFMCGGNNAA00&SELECT=S.sh%7c&Expand=1&Main+Search+Page=Main+Search+Page) | [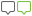](http://ovidsp.tx.ovid.com/sp-3.27.2b/ovidweb.cgi?&S=FOPEFPHDIGDDOIIENCFKDFMCGGNNAA00&R=11&Search+Annotations+Options=SA) |
|  | 12 | Thumb/ | 8700 | Advanced | [Display Results](http://ovidsp.tx.ovid.com/sp-3.27.2b/ovidweb.cgi?&S=FOPEFPHDIGDDOIIENCFKDFMCGGNNAA00&SELECT=S.sh%7c&R=12&Process+Action=display)  [More](http://ovidsp.tx.ovid.com/sp-3.27.2b/ovidweb.cgi?&S=FOPEFPHDIGDDOIIENCFKDFMCGGNNAA00&SELECT=S.sh%7c&Expand=1&Main+Search+Page=Main+Search+Page) | [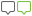](http://ovidsp.tx.ovid.com/sp-3.27.2b/ovidweb.cgi?&S=FOPEFPHDIGDDOIIENCFKDFMCGGNNAA00&R=12&Search+Annotations+Options=SA) |
|  | 13 | thumb*.ti,ab. | 18175 | Advanced | [Display Results](http://ovidsp.tx.ovid.com/sp-3.27.2b/ovidweb.cgi?&S=FOPEFPHDIGDDOIIENCFKDFMCGGNNAA00&SELECT=S.sh%7c&R=13&Process+Action=display)  [More](http://ovidsp.tx.ovid.com/sp-3.27.2b/ovidweb.cgi?&S=FOPEFPHDIGDDOIIENCFKDFMCGGNNAA00&SELECT=S.sh%7c&Expand=1&Main+Search+Page=Main+Search+Page) | [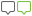](http://ovidsp.tx.ovid.com/sp-3.27.2b/ovidweb.cgi?&S=FOPEFPHDIGDDOIIENCFKDFMCGGNNAA00&R=13&Search+Annotations+Options=SA) |
|  | 14 | pollex.ti,ab. | 73 | Advanced | [Display Results](http://ovidsp.tx.ovid.com/sp-3.27.2b/ovidweb.cgi?&S=FOPEFPHDIGDDOIIENCFKDFMCGGNNAA00&SELECT=S.sh%7c&R=14&Process+Action=display)  [More](http://ovidsp.tx.ovid.com/sp-3.27.2b/ovidweb.cgi?&S=FOPEFPHDIGDDOIIENCFKDFMCGGNNAA00&SELECT=S.sh%7c&Expand=1&Main+Search+Page=Main+Search+Page) | [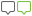](http://ovidsp.tx.ovid.com/sp-3.27.2b/ovidweb.cgi?&S=FOPEFPHDIGDDOIIENCFKDFMCGGNNAA00&R=14&Search+Annotations+Options=SA) |
|  | 15 | (first digit* adj3 hand*).ti,ab. | 9 | Advanced | [Display Results](http://ovidsp.tx.ovid.com/sp-3.27.2b/ovidweb.cgi?&S=FOPEFPHDIGDDOIIENCFKDFMCGGNNAA00&SELECT=S.sh%7c&R=15&Process+Action=display)  [More](http://ovidsp.tx.ovid.com/sp-3.27.2b/ovidweb.cgi?&S=FOPEFPHDIGDDOIIENCFKDFMCGGNNAA00&SELECT=S.sh%7c&Expand=1&Main+Search+Page=Main+Search+Page) | [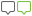](http://ovidsp.tx.ovid.com/sp-3.27.2b/ovidweb.cgi?&S=FOPEFPHDIGDDOIIENCFKDFMCGGNNAA00&R=15&Search+Annotations+Options=SA) |
|  | 16 | (1st digit* adj3 hand*).ti,ab. | 2 | Advanced | [Display Results](http://ovidsp.tx.ovid.com/sp-3.27.2b/ovidweb.cgi?&S=FOPEFPHDIGDDOIIENCFKDFMCGGNNAA00&SELECT=S.sh%7c&R=16&Process+Action=display)  [More](http://ovidsp.tx.ovid.com/sp-3.27.2b/ovidweb.cgi?&S=FOPEFPHDIGDDOIIENCFKDFMCGGNNAA00&SELECT=S.sh%7c&Expand=1&Main+Search+Page=Main+Search+Page) | [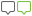](http://ovidsp.tx.ovid.com/sp-3.27.2b/ovidweb.cgi?&S=FOPEFPHDIGDDOIIENCFKDFMCGGNNAA00&R=16&Search+Annotations+Options=SA) |
|  | 17 | (preaxial* adj3 hand*).ti,ab. | 38 | Advanced | [Display Results](http://ovidsp.tx.ovid.com/sp-3.27.2b/ovidweb.cgi?&S=FOPEFPHDIGDDOIIENCFKDFMCGGNNAA00&SELECT=S.sh%7c&R=17&Process+Action=display)  [More](http://ovidsp.tx.ovid.com/sp-3.27.2b/ovidweb.cgi?&S=FOPEFPHDIGDDOIIENCFKDFMCGGNNAA00&SELECT=S.sh%7c&Expand=1&Main+Search+Page=Main+Search+Page) | [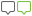](http://ovidsp.tx.ovid.com/sp-3.27.2b/ovidweb.cgi?&S=FOPEFPHDIGDDOIIENCFKDFMCGGNNAA00&R=17&Search+Annotations+Options=SA) |
|  | 18 | (pre axial* adj3 hand*).ti,ab. | 3 | Advanced | [Display Results](http://ovidsp.tx.ovid.com/sp-3.27.2b/ovidweb.cgi?&S=FOPEFPHDIGDDOIIENCFKDFMCGGNNAA00&SELECT=S.sh%7c&R=18&Process+Action=display)  [More](http://ovidsp.tx.ovid.com/sp-3.27.2b/ovidweb.cgi?&S=FOPEFPHDIGDDOIIENCFKDFMCGGNNAA00&SELECT=S.sh%7c&Expand=1&Main+Search+Page=Main+Search+Page) | [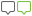](http://ovidsp.tx.ovid.com/sp-3.27.2b/ovidweb.cgi?&S=FOPEFPHDIGDDOIIENCFKDFMCGGNNAA00&R=18&Search+Annotations+Options=SA) |
|  | 19 | 11 or 13 or 14 or 15 or 16 or 17 or 18 | 18827 | Advanced | [Display Results](http://ovidsp.tx.ovid.com/sp-3.27.2b/ovidweb.cgi?&S=FOPEFPHDIGDDOIIENCFKDFMCGGNNAA00&SELECT=S.sh%7c&R=19&Process+Action=display)  [More](http://ovidsp.tx.ovid.com/sp-3.27.2b/ovidweb.cgi?&S=FOPEFPHDIGDDOIIENCFKDFMCGGNNAA00&SELECT=S.sh%7c&Expand=1&Main+Search+Page=Main+Search+Page) | [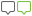](http://ovidsp.tx.ovid.com/sp-3.27.2b/ovidweb.cgi?&S=FOPEFPHDIGDDOIIENCFKDFMCGGNNAA00&R=19&Search+Annotations+Options=SA) |
|  | 20 | 12 or 13 or 14 or 15 or 16 or 17 or 18 | 20178 | Advanced | [Display Results](http://ovidsp.tx.ovid.com/sp-3.27.2b/ovidweb.cgi?&S=FOPEFPHDIGDDOIIENCFKDFMCGGNNAA00&SELECT=S.sh%7c&R=20&Process+Action=display)  [More](http://ovidsp.tx.ovid.com/sp-3.27.2b/ovidweb.cgi?&S=FOPEFPHDIGDDOIIENCFKDFMCGGNNAA00&SELECT=S.sh%7c&Expand=1&Main+Search+Page=Main+Search+Page) | [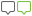](http://ovidsp.tx.ovid.com/sp-3.27.2b/ovidweb.cgi?&S=FOPEFPHDIGDDOIIENCFKDFMCGGNNAA00&R=20&Search+Annotations+Options=SA) |
|  | 21 | 2 or 3 or 4 or 6 or 7 or 8 or 10 | 35847 | Advanced | [Display Results](http://ovidsp.tx.ovid.com/sp-3.27.2b/ovidweb.cgi?&S=FOPEFPHDIGDDOIIENCFKDFMCGGNNAA00&SELECT=S.sh%7c&R=21&Process+Action=display)  [More](http://ovidsp.tx.ovid.com/sp-3.27.2b/ovidweb.cgi?&S=FOPEFPHDIGDDOIIENCFKDFMCGGNNAA00&SELECT=S.sh%7c&Expand=1&Main+Search+Page=Main+Search+Page) | [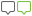](http://ovidsp.tx.ovid.com/sp-3.27.2b/ovidweb.cgi?&S=FOPEFPHDIGDDOIIENCFKDFMCGGNNAA00&R=21&Search+Annotations+Options=SA) |
|  | 22 | 2 or 3 or 5 or 6 or 7 or 9 or 10 | 38311 | Advanced | [Display Results](http://ovidsp.tx.ovid.com/sp-3.27.2b/ovidweb.cgi?&S=FOPEFPHDIGDDOIIENCFKDFMCGGNNAA00&SELECT=S.sh%7c&R=22&Process+Action=display)  [More](http://ovidsp.tx.ovid.com/sp-3.27.2b/ovidweb.cgi?&S=FOPEFPHDIGDDOIIENCFKDFMCGGNNAA00&SELECT=S.sh%7c&Expand=1&Main+Search+Page=Main+Search+Page) | [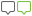](http://ovidsp.tx.ovid.com/sp-3.27.2b/ovidweb.cgi?&S=FOPEFPHDIGDDOIIENCFKDFMCGGNNAA00&R=22&Search+Annotations+Options=SA) |
|  | 23 | 19 and 21 | 1419 | Advanced | [Display Results](http://ovidsp.tx.ovid.com/sp-3.27.2b/ovidweb.cgi?&S=FOPEFPHDIGDDOIIENCFKDFMCGGNNAA00&SELECT=S.sh%7c&R=23&Process+Action=display)  [More](http://ovidsp.tx.ovid.com/sp-3.27.2b/ovidweb.cgi?&S=FOPEFPHDIGDDOIIENCFKDFMCGGNNAA00&SELECT=S.sh%7c&Expand=1&Main+Search+Page=Main+Search+Page) | [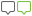](http://ovidsp.tx.ovid.com/sp-3.27.2b/ovidweb.cgi?&S=FOPEFPHDIGDDOIIENCFKDFMCGGNNAA00&R=23&Search+Annotations+Options=SA) |
|  | 24 | 20 and 22 | 1672 | Advanced | [Display Results](http://ovidsp.tx.ovid.com/sp-3.27.2b/ovidweb.cgi?&S=FOPEFPHDIGDDOIIENCFKDFMCGGNNAA00&SELECT=S.sh%7c&R=24&Process+Action=display)  [More](http://ovidsp.tx.ovid.com/sp-3.27.2b/ovidweb.cgi?&S=FOPEFPHDIGDDOIIENCFKDFMCGGNNAA00&SELECT=S.sh%7c&Expand=1&Main+Search+Page=Main+Search+Page) | [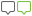](http://ovidsp.tx.ovid.com/sp-3.27.2b/ovidweb.cgi?&S=FOPEFPHDIGDDOIIENCFKDFMCGGNNAA00&R=24&Search+Annotations+Options=SA) |
|  | 25 | gamekeepers thumb*.ti,ab. | 69 | Advanced | [Display Results](http://ovidsp.tx.ovid.com/sp-3.27.2b/ovidweb.cgi?&S=FOPEFPHDIGDDOIIENCFKDFMCGGNNAA00&SELECT=S.sh%7c&R=25&Process+Action=display)  [More](http://ovidsp.tx.ovid.com/sp-3.27.2b/ovidweb.cgi?&S=FOPEFPHDIGDDOIIENCFKDFMCGGNNAA00&SELECT=S.sh%7c&Expand=1&Main+Search+Page=Main+Search+Page) | [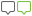](http://ovidsp.tx.ovid.com/sp-3.27.2b/ovidweb.cgi?&S=FOPEFPHDIGDDOIIENCFKDFMCGGNNAA00&R=25&Search+Annotations+Options=SA) |
|  | 26 | skiers thumb*.ti,ab. | 61 | Advanced | [Display Results](http://ovidsp.tx.ovid.com/sp-3.27.2b/ovidweb.cgi?&S=FOPEFPHDIGDDOIIENCFKDFMCGGNNAA00&SELECT=S.sh%7c&R=26&Process+Action=display)  [More](http://ovidsp.tx.ovid.com/sp-3.27.2b/ovidweb.cgi?&S=FOPEFPHDIGDDOIIENCFKDFMCGGNNAA00&SELECT=S.sh%7c&Expand=1&Main+Search+Page=Main+Search+Page) | [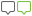](http://ovidsp.tx.ovid.com/sp-3.27.2b/ovidweb.cgi?&S=FOPEFPHDIGDDOIIENCFKDFMCGGNNAA00&R=26&Search+Annotations+Options=SA) |
|  | 27 | 1 or 23 or 25 or 26 | 1459 | Advanced | [Display Results](http://ovidsp.tx.ovid.com/sp-3.27.2b/ovidweb.cgi?&S=FOPEFPHDIGDDOIIENCFKDFMCGGNNAA00&SELECT=S.sh%7c&R=27&Process+Action=display)  [More](http://ovidsp.tx.ovid.com/sp-3.27.2b/ovidweb.cgi?&S=FOPEFPHDIGDDOIIENCFKDFMCGGNNAA00&SELECT=S.sh%7c&Expand=1&Main+Search+Page=Main+Search+Page) | [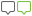](http://ovidsp.tx.ovid.com/sp-3.27.2b/ovidweb.cgi?&S=FOPEFPHDIGDDOIIENCFKDFMCGGNNAA00&R=27&Search+Annotations+Options=SA) |
|  | 28 | 1 or 24 or 25 or 26 | 1707 | Advanced | [Display Results](http://ovidsp.tx.ovid.com/sp-3.27.2b/ovidweb.cgi?&S=FOPEFPHDIGDDOIIENCFKDFMCGGNNAA00&SELECT=S.sh%7c&R=28&Process+Action=display)  [More](http://ovidsp.tx.ovid.com/sp-3.27.2b/ovidweb.cgi?&S=FOPEFPHDIGDDOIIENCFKDFMCGGNNAA00&SELECT=S.sh%7c&Expand=1&Main+Search+Page=Main+Search+Page) | [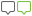](http://ovidsp.tx.ovid.com/sp-3.27.2b/ovidweb.cgi?&S=FOPEFPHDIGDDOIIENCFKDFMCGGNNAA00&R=28&Search+Annotations+Options=SA) |
|  | 29 | randomized controlled trial/ | 485121 | Advanced | [Display Results](http://ovidsp.tx.ovid.com/sp-3.27.2b/ovidweb.cgi?&S=FOPEFPHDIGDDOIIENCFKDFMCGGNNAA00&SELECT=S.sh%7c&R=29&Process+Action=display)  [More](http://ovidsp.tx.ovid.com/sp-3.27.2b/ovidweb.cgi?&S=FOPEFPHDIGDDOIIENCFKDFMCGGNNAA00&SELECT=S.sh%7c&Expand=1&Main+Search+Page=Main+Search+Page) | [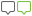](http://ovidsp.tx.ovid.com/sp-3.27.2b/ovidweb.cgi?&S=FOPEFPHDIGDDOIIENCFKDFMCGGNNAA00&R=29&Search+Annotations+Options=SA) |
|  | 30 | single blind procedure/ or double blind procedure/ | 174277 | Advanced | [Display Results](http://ovidsp.tx.ovid.com/sp-3.27.2b/ovidweb.cgi?&S=FOPEFPHDIGDDOIIENCFKDFMCGGNNAA00&SELECT=S.sh%7c&R=30&Process+Action=display)  [More](http://ovidsp.tx.ovid.com/sp-3.27.2b/ovidweb.cgi?&S=FOPEFPHDIGDDOIIENCFKDFMCGGNNAA00&SELECT=S.sh%7c&Expand=1&Main+Search+Page=Main+Search+Page) | [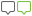](http://ovidsp.tx.ovid.com/sp-3.27.2b/ovidweb.cgi?&S=FOPEFPHDIGDDOIIENCFKDFMCGGNNAA00&R=30&Search+Annotations+Options=SA) |
|  | 31 | crossover procedure/ | 54107 | Advanced | [Display Results](http://ovidsp.tx.ovid.com/sp-3.27.2b/ovidweb.cgi?&S=FOPEFPHDIGDDOIIENCFKDFMCGGNNAA00&SELECT=S.sh%7c&R=31&Process+Action=display)  [More](http://ovidsp.tx.ovid.com/sp-3.27.2b/ovidweb.cgi?&S=FOPEFPHDIGDDOIIENCFKDFMCGGNNAA00&SELECT=S.sh%7c&Expand=1&Main+Search+Page=Main+Search+Page) | [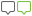](http://ovidsp.tx.ovid.com/sp-3.27.2b/ovidweb.cgi?&S=FOPEFPHDIGDDOIIENCFKDFMCGGNNAA00&R=31&Search+Annotations+Options=SA) |
|  | 32 | random*.tw. | 1261288 | Advanced | [Display Results](http://ovidsp.tx.ovid.com/sp-3.27.2b/ovidweb.cgi?&S=FOPEFPHDIGDDOIIENCFKDFMCGGNNAA00&SELECT=S.sh%7c&R=32&Process+Action=display)  [More](http://ovidsp.tx.ovid.com/sp-3.27.2b/ovidweb.cgi?&S=FOPEFPHDIGDDOIIENCFKDFMCGGNNAA00&SELECT=S.sh%7c&Expand=1&Main+Search+Page=Main+Search+Page) | [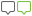](http://ovidsp.tx.ovid.com/sp-3.27.2b/ovidweb.cgi?&S=FOPEFPHDIGDDOIIENCFKDFMCGGNNAA00&R=32&Search+Annotations+Options=SA) |
|  | 33 | (random or ((singl* or doubl*) adj (blind* or mask*)) or crossover or cross over or factorial* or latin square or assign* or allocat* or volunteer*).ti,ab. | 1142338 | Advanced | [Display Results](http://ovidsp.tx.ovid.com/sp-3.27.2b/ovidweb.cgi?&S=FOPEFPHDIGDDOIIENCFKDFMCGGNNAA00&SELECT=S.sh%7c&R=33&Process+Action=display)  [More](http://ovidsp.tx.ovid.com/sp-3.27.2b/ovidweb.cgi?&S=FOPEFPHDIGDDOIIENCFKDFMCGGNNAA00&SELECT=S.sh%7c&Expand=1&Main+Search+Page=Main+Search+Page) | [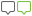](http://ovidsp.tx.ovid.com/sp-3.27.2b/ovidweb.cgi?&S=FOPEFPHDIGDDOIIENCFKDFMCGGNNAA00&R=33&Search+Annotations+Options=SA) |
|  | 34 | 29 or 30 or 31 or 32 or 33 | 1900737 | Advanced | [Display Results](http://ovidsp.tx.ovid.com/sp-3.27.2b/ovidweb.cgi?&S=FOPEFPHDIGDDOIIENCFKDFMCGGNNAA00&SELECT=S.sh%7c&R=34&Process+Action=display)  [More](http://ovidsp.tx.ovid.com/sp-3.27.2b/ovidweb.cgi?&S=FOPEFPHDIGDDOIIENCFKDFMCGGNNAA00&SELECT=S.sh%7c&Expand=1&Main+Search+Page=Main+Search+Page) | [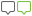](http://ovidsp.tx.ovid.com/sp-3.27.2b/ovidweb.cgi?&S=FOPEFPHDIGDDOIIENCFKDFMCGGNNAA00&R=34&Search+Annotations+Options=SA) |
|  | 35 | (exp animals/ or nonhuman/) not human/ | 6336199 | Advanced | [Display Results](http://ovidsp.tx.ovid.com/sp-3.27.2b/ovidweb.cgi?&S=FOPEFPHDIGDDOIIENCFKDFMCGGNNAA00&SELECT=S.sh%7c&R=35&Process+Action=display)  [More](http://ovidsp.tx.ovid.com/sp-3.27.2b/ovidweb.cgi?&S=FOPEFPHDIGDDOIIENCFKDFMCGGNNAA00&SELECT=S.sh%7c&Expand=1&Main+Search+Page=Main+Search+Page) | [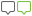](http://ovidsp.tx.ovid.com/sp-3.27.2b/ovidweb.cgi?&S=FOPEFPHDIGDDOIIENCFKDFMCGGNNAA00&R=35&Search+Annotations+Options=SA) |
|  | 36 | 34 not 35 | 1655872 | Advanced | [Display Results](http://ovidsp.tx.ovid.com/sp-3.27.2b/ovidweb.cgi?&S=FOPEFPHDIGDDOIIENCFKDFMCGGNNAA00&SELECT=S.sh%7c&R=36&Process+Action=display)  [More](http://ovidsp.tx.ovid.com/sp-3.27.2b/ovidweb.cgi?&S=FOPEFPHDIGDDOIIENCFKDFMCGGNNAA00&SELECT=S.sh%7c&Expand=1&Main+Search+Page=Main+Search+Page) | [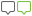](http://ovidsp.tx.ovid.com/sp-3.27.2b/ovidweb.cgi?&S=FOPEFPHDIGDDOIIENCFKDFMCGGNNAA00&R=36&Search+Annotations+Options=SA) |
|  | 37 | 27 and 36 | 56 | Advanced | [Display Results](http://ovidsp.tx.ovid.com/sp-3.27.2b/ovidweb.cgi?&S=FOPEFPHDIGDDOIIENCFKDFMCGGNNAA00&SELECT=S.sh%7c&R=37&Process+Action=display)  [More](http://ovidsp.tx.ovid.com/sp-3.27.2b/ovidweb.cgi?&S=FOPEFPHDIGDDOIIENCFKDFMCGGNNAA00&SELECT=S.sh%7c&Expand=1&Main+Search+Page=Main+Search+Page) | [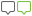](http://ovidsp.tx.ovid.com/sp-3.27.2b/ovidweb.cgi?&S=FOPEFPHDIGDDOIIENCFKDFMCGGNNAA00&R=37&Search+Annotations+Options=SA) |
|  | 38 | 28 and 36 | 64 | Advanced | [Display Results](http://ovidsp.tx.ovid.com/sp-3.27.2b/ovidweb.cgi?&S=FOPEFPHDIGDDOIIENCFKDFMCGGNNAA00&SELECT=S.sh%7c&R=38&Process+Action=display)  [More](http://ovidsp.tx.ovid.com/sp-3.27.2b/ovidweb.cgi?&S=FOPEFPHDIGDDOIIENCFKDFMCGGNNAA00&SELECT=S.sh%7c&Expand=1&Main+Search+Page=Main+Search+Page) | [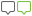](http://ovidsp.tx.ovid.com/sp-3.27.2b/ovidweb.cgi?&S=FOPEFPHDIGDDOIIENCFKDFMCGGNNAA00&R=38&Search+Annotations+Options=SA) |
|  | | |  |  |  |  |

Combine with:

|  |  |
| --- | --- |

**CINAHL** (31/02/18)

| **Search Terms** | **Search Options** | **Actions** |  |  |
| --- | --- | --- | --- | --- |
|  | S22 | S1 OR S18 OR S19 OR S20 | Limiters - Clinical Queries: Therapy - High Specificity  Search modes - Boolean/Phrase | [View Results](javascript:__doPostBack('ctl00$ctl00$MainContentArea$MainContentArea$historyControl$HistoryRepeater$ctl00$linkResults','')) (3)  [View Details](javascript:showShDetails(%22ctl00_ctl00_MainContentArea_MainContentArea_historyControl_ctrlPopup%22,%20%22S22%22);)  [Edit](http://web.b.ebscohost.com/Legacy/Views/UserControls/EHOST/) |
|  | S21 | S1 OR S18 OR S19 OR S20 | Search modes - Boolean/Phrase | [View Results](javascript:__doPostBack('ctl00$ctl00$MainContentArea$MainContentArea$historyControl$HistoryRepeater$ctl01$linkResults','')) (138)  [View Details](javascript:showShDetails(%22ctl00_ctl00_MainContentArea_MainContentArea_historyControl_ctrlPopup%22,%20%22S21%22);)  [Edit](http://web.b.ebscohost.com/Legacy/Views/UserControls/EHOST/) |
|  | S20 | TI "skiers thumb*" OR AB "skiers thumb*" | Search modes - Boolean/Phrase | [View Results](javascript:__doPostBack('ctl00$ctl00$MainContentArea$MainContentArea$historyControl$HistoryRepeater$ctl02$linkResults','')) (8)  [View Details](javascript:showShDetails(%22ctl00_ctl00_MainContentArea_MainContentArea_historyControl_ctrlPopup%22,%20%22S20%22);)  [Edit](http://web.b.ebscohost.com/Legacy/Views/UserControls/EHOST/) |
|  | S19 | TI "gamekeepers thumb*" OR AB "gamekeepers thumb*" | Search modes - Boolean/Phrase | [View Results](javascript:__doPostBack('ctl00$ctl00$MainContentArea$MainContentArea$historyControl$HistoryRepeater$ctl03$linkResults','')) (2)  [View Details](javascript:showShDetails(%22ctl00_ctl00_MainContentArea_MainContentArea_historyControl_ctrlPopup%22,%20%22S19%22);)  [Edit](http://web.b.ebscohost.com/Legacy/Views/UserControls/EHOST/) |
|  | S18 | S16 AND S17 | Search modes - Boolean/Phrase | [View Results](javascript:__doPostBack('ctl00$ctl00$MainContentArea$MainContentArea$historyControl$HistoryRepeater$ctl04$linkResults','')) (133)  [View Details](javascript:showShDetails(%22ctl00_ctl00_MainContentArea_MainContentArea_historyControl_ctrlPopup%22,%20%22S18%22);)  [Edit](http://web.b.ebscohost.com/Legacy/Views/UserControls/EHOST/) |
|  | S17 | S9 OR S10 OR S11 OR S12 OR S13 OR S14 OR S15 | Search modes - Boolean/Phrase | [View Results](javascript:__doPostBack('ctl00$ctl00$MainContentArea$MainContentArea$historyControl$HistoryRepeater$ctl05$linkResults','')) (1,847)  [View Details](javascript:showShDetails(%22ctl00_ctl00_MainContentArea_MainContentArea_historyControl_ctrlPopup%22,%20%22S17%22);)  [Edit](http://web.b.ebscohost.com/Legacy/Views/UserControls/EHOST/) |
|  | S16 | S2 OR S3 OR S4 OR S5 OR S6 OR S7 OR S8 | Search modes - Boolean/Phrase | [View Results](javascript:__doPostBack('ctl00$ctl00$MainContentArea$MainContentArea$historyControl$HistoryRepeater$ctl06$linkResults','')) (1,889)  [View Details](javascript:showShDetails(%22ctl00_ctl00_MainContentArea_MainContentArea_historyControl_ctrlPopup%22,%20%22S16%22);)  [Edit](http://web.b.ebscohost.com/Legacy/Views/UserControls/EHOST/) |
|  | S15 | TI "pre axial*" N3 hand* OR AB "pre axial*" N3 hand* | Search modes - Boolean/Phrase | [View Results](javascript:__doPostBack('ctl00$ctl00$MainContentArea$MainContentArea$historyControl$HistoryRepeater$ctl07$linkResults','')) (1)  [View Details](javascript:showShDetails(%22ctl00_ctl00_MainContentArea_MainContentArea_historyControl_ctrlPopup%22,%20%22S15%22);)  [Edit](http://web.b.ebscohost.com/Legacy/Views/UserControls/EHOST/) |
|  | S14 | TI preaxial* N3 hand* OR AB preaxial* N3 hand* | Search modes - Boolean/Phrase | [View Results](javascript:__doPostBack('ctl00$ctl00$MainContentArea$MainContentArea$historyControl$HistoryRepeater$ctl08$linkResults','')) (3)  [View Details](javascript:showShDetails(%22ctl00_ctl00_MainContentArea_MainContentArea_historyControl_ctrlPopup%22,%20%22S14%22);)  [Edit](http://web.b.ebscohost.com/Legacy/Views/UserControls/EHOST/) |
|  | S13 | TI "1st digit*" N3 hand* OR AB "1st digit*" N3 hand* | Search modes - Boolean/Phrase | [View Results](javascript:__doPostBack('ctl00$ctl00$MainContentArea$MainContentArea$historyControl$HistoryRepeater$ctl09$linkResults','')) (625)  [View Details](javascript:showShDetails(%22ctl00_ctl00_MainContentArea_MainContentArea_historyControl_ctrlPopup%22,%20%22S13%22);)  [Edit](http://web.b.ebscohost.com/Legacy/Views/UserControls/EHOST/) |
|  | S12 | TI "first digit*" N3 hand* OR AB "first digit*" N3 hand* | Search modes - Boolean/Phrase | [View Results](javascript:__doPostBack('ctl00$ctl00$MainContentArea$MainContentArea$historyControl$HistoryRepeater$ctl10$linkResults','')) (235)  [View Details](javascript:showShDetails(%22ctl00_ctl00_MainContentArea_MainContentArea_historyControl_ctrlPopup%22,%20%22S12%22);)  [Edit](http://web.b.ebscohost.com/Legacy/Views/UserControls/EHOST/) |
|  | S11 | TI pollex OR AB pollex | Search modes - Boolean/Phrase | [View Results](javascript:__doPostBack('ctl00$ctl00$MainContentArea$MainContentArea$historyControl$HistoryRepeater$ctl11$linkResults','')) (0)  [View Details](javascript:showShDetails(%22ctl00_ctl00_MainContentArea_MainContentArea_historyControl_ctrlPopup%22,%20%22S11%22);)  [Edit](http://web.b.ebscohost.com/Legacy/Views/UserControls/EHOST/) |
|  | S10 | TI thumb* OR AB thumb* | Search modes - Boolean/Phrase | [View Results](javascript:__doPostBack('ctl00$ctl00$MainContentArea$MainContentArea$historyControl$HistoryRepeater$ctl12$linkResults','')) (1,656)  [View Details](javascript:showShDetails(%22ctl00_ctl00_MainContentArea_MainContentArea_historyControl_ctrlPopup%22,%20%22S10%22);)  [Edit](http://web.b.ebscohost.com/Legacy/Views/UserControls/EHOST/) |
|  | S9 | (MH "Thumb") | Search modes - Boolean/Phrase | [View Results](javascript:__doPostBack('ctl00$ctl00$MainContentArea$MainContentArea$historyControl$HistoryRepeater$ctl13$linkResults','')) (660)  [View Details](javascript:showShDetails(%22ctl00_ctl00_MainContentArea_MainContentArea_historyControl_ctrlPopup%22,%20%22S9%22);)  [Edit](http://web.b.ebscohost.com/Legacy/Views/UserControls/EHOST/) |
|  | S8 | TI metacarpal phalan* N3 joint* OR AB metacarpal phalan* N3 joint* | Search modes - Boolean/Phrase | [View Results](javascript:__doPostBack('ctl00$ctl00$MainContentArea$MainContentArea$historyControl$HistoryRepeater$ctl14$linkResults','')) (17)  [View Details](javascript:showShDetails(%22ctl00_ctl00_MainContentArea_MainContentArea_historyControl_ctrlPopup%22,%20%22S8%22);)  [Edit](http://web.b.ebscohost.com/Legacy/Views/UserControls/EHOST/) |
|  | S7 | TI metacarpophalan* N3 joint* OR AB metacarpophalan* N3 joint* | Search modes - Boolean/Phrase | [View Results](javascript:__doPostBack('ctl00$ctl00$MainContentArea$MainContentArea$historyControl$HistoryRepeater$ctl15$linkResults','')) (338)  [View Details](javascript:showShDetails(%22ctl00_ctl00_MainContentArea_MainContentArea_historyControl_ctrlPopup%22,%20%22S7%22);)  [Edit](http://web.b.ebscohost.com/Legacy/Views/UserControls/EHOST/) |
|  | S6 | TI mcp OR AB mcp | Search modes - Boolean/Phrase | [View Results](javascript:__doPostBack('ctl00$ctl00$MainContentArea$MainContentArea$historyControl$HistoryRepeater$ctl16$linkResults','')) (881)  [View Details](javascript:showShDetails(%22ctl00_ctl00_MainContentArea_MainContentArea_historyControl_ctrlPopup%22,%20%22S6%22);)  [Edit](http://web.b.ebscohost.com/Legacy/Views/UserControls/EHOST/) |
|  | S5 | (MH "Collateral Ligaments") | Search modes - Boolean/Phrase | [View Results](javascript:__doPostBack('ctl00$ctl00$MainContentArea$MainContentArea$historyControl$HistoryRepeater$ctl17$linkResults','')) (432)  [View Details](javascript:showShDetails(%22ctl00_ctl00_MainContentArea_MainContentArea_historyControl_ctrlPopup%22,%20%22S5%22);)  [Edit](http://web.b.ebscohost.com/Legacy/Views/UserControls/EHOST/) |
|  | S4 | (MH "Metacarpophalangeal Joint") | Search modes - Boolean/Phrase | [View Results](javascript:__doPostBack('ctl00$ctl00$MainContentArea$MainContentArea$historyControl$HistoryRepeater$ctl18$linkResults','')) (285)  [View Details](javascript:showShDetails(%22ctl00_ctl00_MainContentArea_MainContentArea_historyControl_ctrlPopup%22,%20%22S4%22);)  [Edit](http://web.b.ebscohost.com/Legacy/Views/UserControls/EHOST/) |
|  | S3 | TI ucl OR AB ucl | Search modes - Boolean/Phrase | [View Results](javascript:__doPostBack('ctl00$ctl00$MainContentArea$MainContentArea$historyControl$HistoryRepeater$ctl19$linkResults','')) (148)  [View Details](javascript:showShDetails(%22ctl00_ctl00_MainContentArea_MainContentArea_historyControl_ctrlPopup%22,%20%22S3%22);)  [Edit](http://web.b.ebscohost.com/Legacy/Views/UserControls/EHOST/) |
|  | S2 | TI ulnar N3 collateral ligament* OR AB ulnar N3 collateral ligament* | Search modes - Boolean/Phrase | [View Results](javascript:__doPostBack('ctl00$ctl00$MainContentArea$MainContentArea$historyControl$HistoryRepeater$ctl20$linkResults','')) (237)  [View Details](javascript:showShDetails(%22ctl00_ctl00_MainContentArea_MainContentArea_historyControl_ctrlPopup%22,%20%22S2%22);)  [Edit](http://web.b.ebscohost.com/Legacy/Views/UserControls/EHOST/) |
|  | S1 | (MH "Ulnar Collateral Ligament Injury of the Thumb") | Search modes - Boolean/Phrase | [View Results](javascript:__doPostBack('ctl00$ctl00$MainContentArea$MainContentArea$historyControl$HistoryRepeater$ctl21$linkResults','')) (6)  [View Details](javascript:showShDetails(%22ctl00_ctl00_MainContentArea_MainContentArea_historyControl_ctrlPopup%22,%20%22S1%22);)  [Edit](http://web.b.ebscohost.com/Legacy/Views/UserControls/EHOST/) |

**SPORTDiscus** (31/02/18)

| **Search Terms** | **Search Options** | **Actions** |  |  |
| --- | --- | --- | --- | --- |
|  | S19 | S17 AND S18 | Search modes - Boolean/Phrase | [View Results](javascript:__doPostBack('ctl00$ctl00$FindField$FindField$historyControl$HistoryRepeater$ctl00$linkResults','')) (9)  [View Details](javascript:showShDetails(%22ctl00_ctl00_FindField_FindField_historyControl_ctrlPopup%22,%20%22S19%22);)  [Edit](http://web.b.ebscohost.com/Legacy/Views/UserControls/Ehost/) |
|  | S18 | TI ( random* OR trial* OR allocat* OR crossover OR cross-over OR groups ) OR AB ( random* OR trial* OR allocat* OR crossover OR cross-over OR groups ) | Search modes - Boolean/Phrase | [View Results](javascript:__doPostBack('ctl00$ctl00$FindField$FindField$historyControl$HistoryRepeater$ctl01$linkResults','')) (178,670)  [View Details](javascript:showShDetails(%22ctl00_ctl00_FindField_FindField_historyControl_ctrlPopup%22,%20%22S18%22);)  [Edit](http://web.b.ebscohost.com/Legacy/Views/UserControls/Ehost/) |
|  | S17 | S14 OR S15 OR S16 | Search modes - Boolean/Phrase | [View Results](javascript:__doPostBack('ctl00$ctl00$FindField$FindField$historyControl$HistoryRepeater$ctl02$linkResults','')) (99)  [View Details](javascript:showShDetails(%22ctl00_ctl00_FindField_FindField_historyControl_ctrlPopup%22,%20%22S17%22);)  [Edit](http://web.b.ebscohost.com/Legacy/Views/UserControls/Ehost/) |
|  | S16 | TI "gamekeepers thumb*" OR AB "gamekeepers thumb*" | Search modes - Boolean/Phrase | [View Results](javascript:__doPostBack('ctl00$ctl00$FindField$FindField$historyControl$HistoryRepeater$ctl03$linkResults','')) (0)  [View Details](javascript:showShDetails(%22ctl00_ctl00_FindField_FindField_historyControl_ctrlPopup%22,%20%22S16%22);)  [Edit](http://web.b.ebscohost.com/Legacy/Views/UserControls/Ehost/) |
|  | S15 | TI "skiers thumb*" OR AB "skiers thumb*" | Search modes - Boolean/Phrase | [View Results](javascript:__doPostBack('ctl00$ctl00$FindField$FindField$historyControl$HistoryRepeater$ctl04$linkResults','')) (2)  [View Details](javascript:showShDetails(%22ctl00_ctl00_FindField_FindField_historyControl_ctrlPopup%22,%20%22S15%22);)  [Edit](http://web.b.ebscohost.com/Legacy/Views/UserControls/Ehost/) |
|  | S14 | S12 AND S13 | Search modes - Boolean/Phrase | [View Results](javascript:__doPostBack('ctl00$ctl00$FindField$FindField$historyControl$HistoryRepeater$ctl05$linkResults','')) (97)  [View Details](javascript:showShDetails(%22ctl00_ctl00_FindField_FindField_historyControl_ctrlPopup%22,%20%22S14%22);)  [Edit](http://web.b.ebscohost.com/Legacy/Views/UserControls/Ehost/) |
|  | S13 | S6 OR S7 OR S8 OR S9 OR S10 OR S11 | Search modes - Boolean/Phrase | [View Results](javascript:__doPostBack('ctl00$ctl00$FindField$FindField$historyControl$HistoryRepeater$ctl06$linkResults','')) (1,528)  [View Details](javascript:showShDetails(%22ctl00_ctl00_FindField_FindField_historyControl_ctrlPopup%22,%20%22S13%22);)  [Edit](http://web.b.ebscohost.com/Legacy/Views/UserControls/Ehost/) |
|  | S12 | S1 OR S2 OR S3 OR S4 OR S5 | Search modes - Boolean/Phrase | [View Results](javascript:__doPostBack('ctl00$ctl00$FindField$FindField$historyControl$HistoryRepeater$ctl07$linkResults','')) (742)  [View Details](javascript:showShDetails(%22ctl00_ctl00_FindField_FindField_historyControl_ctrlPopup%22,%20%22S12%22);)  [Edit](http://web.b.ebscohost.com/Legacy/Views/UserControls/Ehost/) |
|  | S11 | TI "pre axial*" N3 hand* OR AB "pre axial*" N3 hand* | Search modes - Boolean/Phrase | [View Results](javascript:__doPostBack('ctl00$ctl00$FindField$FindField$historyControl$HistoryRepeater$ctl08$linkResults','')) (121)  [View Details](javascript:showShDetails(%22ctl00_ctl00_FindField_FindField_historyControl_ctrlPopup%22,%20%22S11%22);)  [Edit](http://web.b.ebscohost.com/Legacy/Views/UserControls/Ehost/) |
|  | S10 | TI preaxial* N3 hand* OR AB preaxial* N3 hand* | Search modes - Boolean/Phrase | [View Results](javascript:__doPostBack('ctl00$ctl00$FindField$FindField$historyControl$HistoryRepeater$ctl09$linkResults','')) (2)  [View Details](javascript:showShDetails(%22ctl00_ctl00_FindField_FindField_historyControl_ctrlPopup%22,%20%22S10%22);)  [Edit](http://web.b.ebscohost.com/Legacy/Views/UserControls/Ehost/) |
|  | S9 | TI "1st digit*" N3 hand* OR AB "1st digit*" N3 hand* | Search modes - Boolean/Phrase | [View Results](javascript:__doPostBack('ctl00$ctl00$FindField$FindField$historyControl$HistoryRepeater$ctl10$linkResults','')) (1)  [View Details](javascript:showShDetails(%22ctl00_ctl00_FindField_FindField_historyControl_ctrlPopup%22,%20%22S9%22);)  [Edit](http://web.b.ebscohost.com/Legacy/Views/UserControls/Ehost/) |
|  | S8 | TI "first digit*" N3 hand* OR AB "first digit*" N3 hand* | Search modes - Boolean/Phrase | [View Results](javascript:__doPostBack('ctl00$ctl00$FindField$FindField$historyControl$HistoryRepeater$ctl11$linkResults','')) (135)  [View Details](javascript:showShDetails(%22ctl00_ctl00_FindField_FindField_historyControl_ctrlPopup%22,%20%22S8%22);)  [Edit](http://web.b.ebscohost.com/Legacy/Views/UserControls/Ehost/) |
|  | S7 | TI pollex OR AB pollex | Search modes - Boolean/Phrase | [View Results](javascript:__doPostBack('ctl00$ctl00$FindField$FindField$historyControl$HistoryRepeater$ctl12$linkResults','')) (3)  [View Details](javascript:showShDetails(%22ctl00_ctl00_FindField_FindField_historyControl_ctrlPopup%22,%20%22S7%22);)  [Edit](http://web.b.ebscohost.com/Legacy/Views/UserControls/Ehost/) |
|  | S6 | TI thumb* OR AB thumb* | Search modes - Boolean/Phrase | [View Results](javascript:__doPostBack('ctl00$ctl00$FindField$FindField$historyControl$HistoryRepeater$ctl13$linkResults','')) (1,527)  [View Details](javascript:showShDetails(%22ctl00_ctl00_FindField_FindField_historyControl_ctrlPopup%22,%20%22S6%22);)  [Edit](http://web.b.ebscohost.com/Legacy/Views/UserControls/Ehost/) |
|  | S5 | TI "metacarpal phalan*" N3 joint* OR AB "metacarpal phalan*" N3 joint* | Search modes - Boolean/Phrase | [View Results](javascript:__doPostBack('ctl00$ctl00$FindField$FindField$historyControl$HistoryRepeater$ctl14$linkResults','')) (9)  [View Details](javascript:showShDetails(%22ctl00_ctl00_FindField_FindField_historyControl_ctrlPopup%22,%20%22S5%22);)  [Edit](http://web.b.ebscohost.com/Legacy/Views/UserControls/Ehost/) |
|  | S4 | TI metacarpophalan* N3 joint* OR AB metacarpophalan* N3 joint* | Search modes - Boolean/Phrase | [View Results](javascript:__doPostBack('ctl00$ctl00$FindField$FindField$historyControl$HistoryRepeater$ctl15$linkResults','')) (149)  [View Details](javascript:showShDetails(%22ctl00_ctl00_FindField_FindField_historyControl_ctrlPopup%22,%20%22S4%22);)  [Edit](http://web.b.ebscohost.com/Legacy/Views/UserControls/Ehost/) |
|  | S3 | TI mcp OR AB mcp | Search modes - Boolean/Phrase | [View Results](javascript:__doPostBack('ctl00$ctl00$FindField$FindField$historyControl$HistoryRepeater$ctl16$linkResults','')) (292)  [View Details](javascript:showShDetails(%22ctl00_ctl00_FindField_FindField_historyControl_ctrlPopup%22,%20%22S3%22);)  [Edit](http://web.b.ebscohost.com/Legacy/Views/UserControls/Ehost/) |
|  | S2 | TI ucl OR AB ucl | Search modes - Boolean/Phrase | [View Results](javascript:__doPostBack('ctl00$ctl00$FindField$FindField$historyControl$HistoryRepeater$ctl17$linkResults','')) (151)  [View Details](javascript:showShDetails(%22ctl00_ctl00_FindField_FindField_historyControl_ctrlPopup%22,%20%22S2%22);)  [Edit](http://web.b.ebscohost.com/Legacy/Views/UserControls/Ehost/) |
|  | S1 | TI ulnar N3 "collateral ligament*" OR AB ulnar N3 "collateral ligament*" | Search modes - Boolean/Phrase | [View Results](javascript:__doPostBack('ctl00$ctl00$FindField$FindField$historyControl$HistoryRepeater$ctl18$linkResults','')) (297)  [View Details](javascript:showShDetails(%22ctl00_ctl00_FindField_FindField_historyControl_ctrlPopup%22,%20%22S1%22);)  [Edit](http://web.b.ebscohost.com/Legacy/Views/UserControls/Ehost/) |
