# Supplementary figures and images for: Therapeutic interventions for acute complete ruptures of the ulnar collateral ligament of the thumb: a systematic review
Source: F1000Res. 2018 Jun 8;7:714. [Version 1] doi: 10.12688/f1000research.15065.1 (PMC6051197; doi:10.12688/f1000research.15065.1)

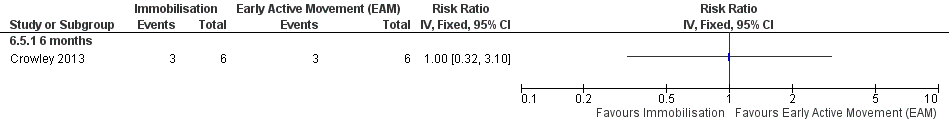

Supplement: Supplementary file 3 [file f1000research-7-16402-s0002.tgz › 144e2dbe-e2d6-4859-aa8f-a4fc5b1e89eb.png]

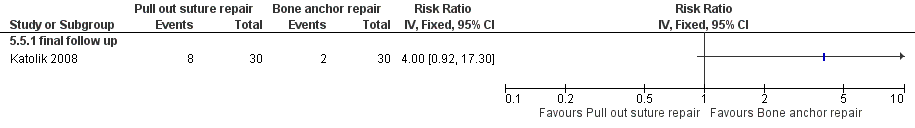

Supplement: Supplementary file 4 [file f1000research-7-16402-s0003.tgz › 62cf2718-76ed-4d9f-8fcc-98a772bd6337.png]

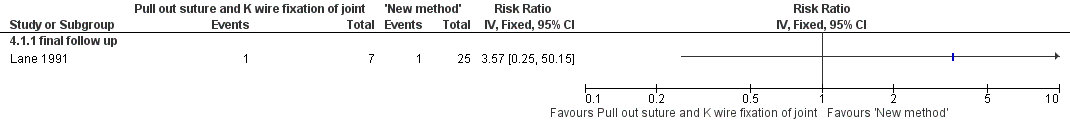

Supplement: Supplementary file 5 [file f1000research-7-16402-s0004.tgz › 128a0476-b091-485a-b737-e3ed2d887da3.png]

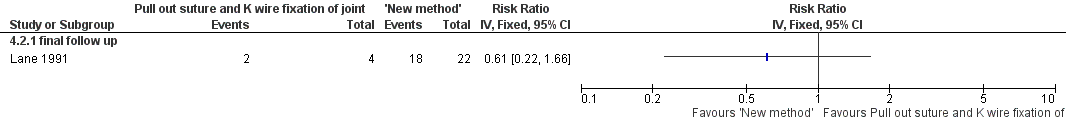

Supplement: Supplementary file 6 [file f1000research-7-16402-s0005.tgz › f7933e89-2c74-4514-9352-04e22868e340.png]

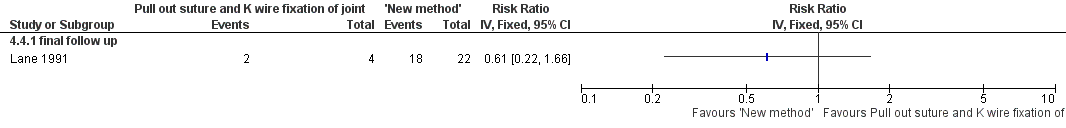

Supplement: Supplementary file 7 [file f1000research-7-16402-s0006.tgz › 9d1a9871-028c-46d2-9f4f-e1a92618d4cb.png]

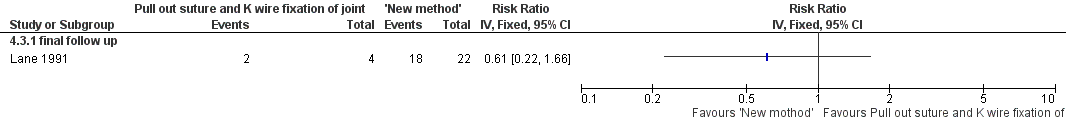

Supplement: Supplementary file 8 [file f1000research-7-16402-s0007.tgz › 9bd72518-70d9-4c36-a645-57318256ebdf.png]

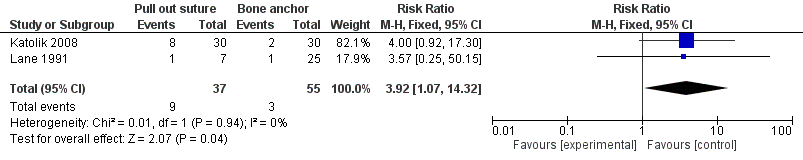

Supplement: Supplementary file 9 [file f1000research-7-16402-s0008.tgz › 2f736a7e-8999-4b4a-a911-b7e3cea36524.png]

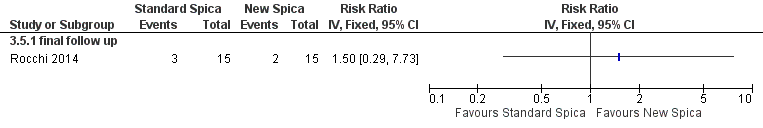

Supplement: Supplementary file 10 [file f1000research-7-16402-s0009.tgz › 9204573f-b3a1-4fac-8707-07e9da73b351.png]

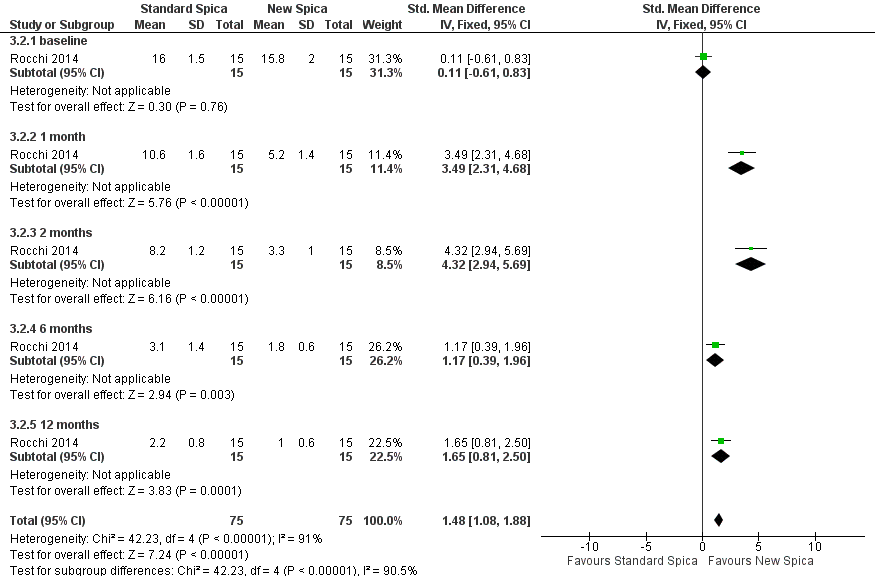

Supplement: Supplementary file 11 [file f1000research-7-16402-s0010.tgz › 946818d4-5725-45b9-862f-6b558bcb7001.png]

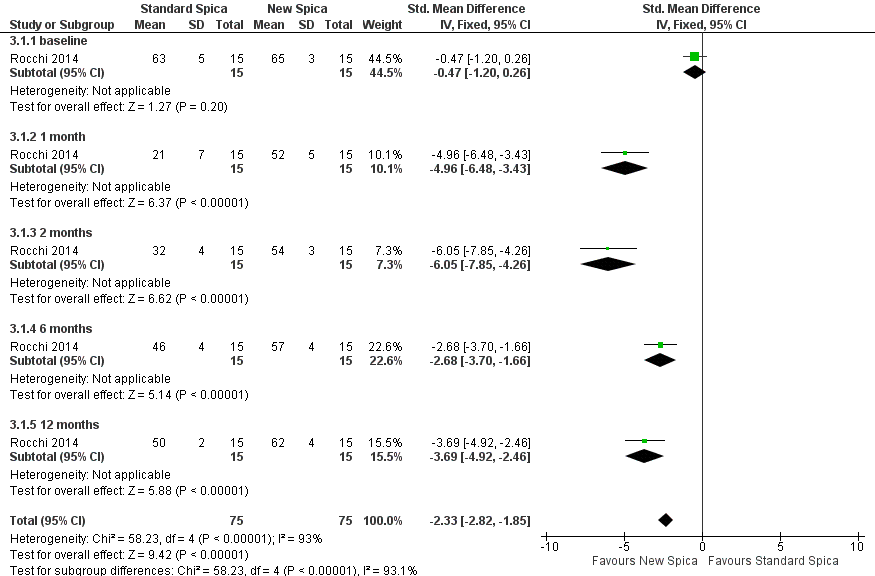

Supplement: Supplementary file 12 [file f1000research-7-16402-s0011.tgz › dd82c298-c03b-4709-b133-6b6843a5761a.png]

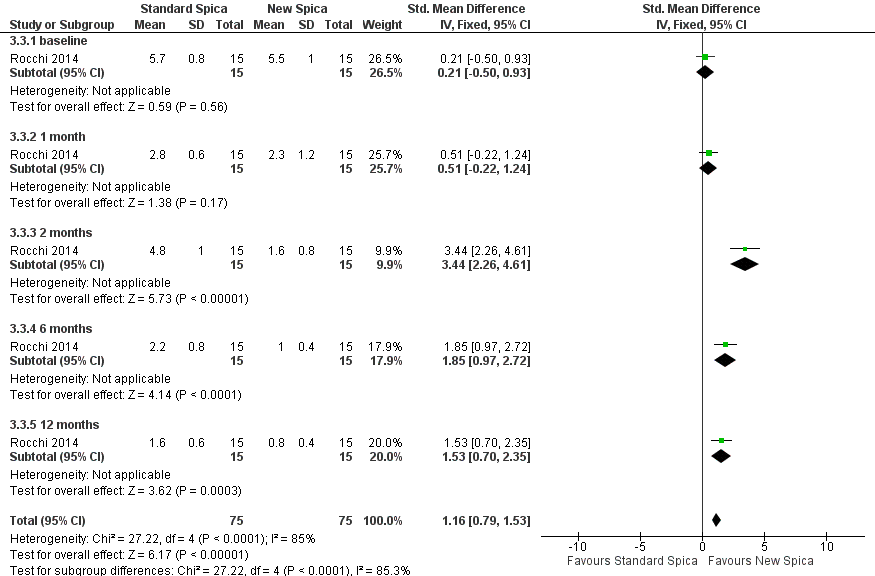

Supplement: Supplementary file 13 [file f1000research-7-16402-s0012.tgz › 9f0135b7-8836-440a-a749-c03595971212.png]

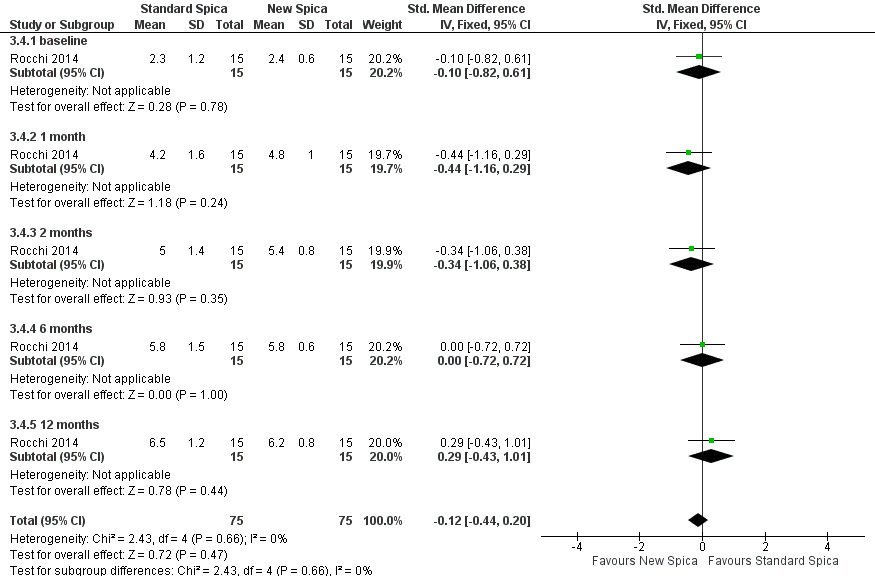

Supplement: Supplementary file 14 [file f1000research-7-16402-s0013.tgz › d6d4ec88-4827-48fa-9b09-2569ea594eba.png]

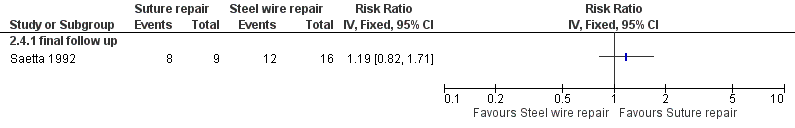

Supplement: Supplementary file 15 [file f1000research-7-16402-s0014.tgz › 2fb8cf73-7629-4e0a-af67-d2e615711743.png]
