# Supplementary material for: Therapeutic interventions for acute complete ruptures of the ulnar collateral ligament of the thumb: a systematic review
Source: F1000Res. 2018 Jun 8;7:714. [Version 1] doi: 10.12688/f1000research.15065.1 (PMC6051197; doi:10.12688/f1000research.15065.1)
Supplement: Peer review history

# Peer review: Referee response for version 1

**DOI:** 10.5256/f1000research.16402.r34857

**Published:** 2018-07-02

**Authors:** Charles Pailthorpe

**Affiliations:** 1 Royal Berkshire Hospital, Reading, UK

The authors aim was to perform a systematic review of the effectiveness of available interventions for acute complete rupture of the ulnar collateral ligament of the thumb in terms of patient-reported outcome measures and to assess the rates of adverse outcomes associated with these interventions.

1. Their methodology appears overall sound, however I have some concerns over their request for additional data from the authors of the selected papers. If this data was not included in the original papers how can it be accepted retrospectively.
2. Overall the authors have achieved their aim of the systematic review however they have added a large amount of extra data particularly concerning diagnosis (MRI and US).

In general I think the paper has merit to be published but in their stated aim the authors should include that they reviewed the literature concerning diagnosis as well.

 11th June 2018: **The status of this report has been updated from ‘Approved with reservations’ to ‘Approved’ in response to the author comments.**

 I thank the authors for their comments and accept that no additional data was either sourced or utilised. Also, I accept their comments on the diagnostic component in the article and accept that on balance it is useful in its contribution to the overall aim.

I have read this submission. I believe that I have an appropriate level of expertise to confirm that it is of an acceptable scientific standard.

# Response

**Published:** 2018-07-02

**Authors:** Benjamin Dean

**Affiliations:** University of Oxford, UK

Many thanks for your comments which we have attempted to address below.

*“Their methodology appears overall sound, however I have some concerns over their request for additional data from the authors of the selected papers. If this data was not included in the original papers how can it be accepted retrospectively.”*

In terms of requesting additional data, we used the approach described and recommended by the Cochrane group ^1^.  No authors were able to provide any additional data, as summarised in Table 2 of our study, therefore no additional data was accepted retrospectively.

*“Overall the authors have achieved their aim of the systematic review however they have added a large amount of extra data particularly concerning diagnosis (MRI and US).”*

We have described some findings relating to diagnosis within the introduction and discussion with the aim of providing context and clinical relevance to the systematic review.  Obviously, this element has not been performed systematically and this was not our intention.  If the reviewer feels the context as regards diagnosis could be better summarised then we are happy to consider any suggestions which may augment the discussion. Should the reviewer feel that the statements regarding radiological investigations are superfluous, then we would be happy to consider excluding them.

**1.**            Young T, Hopewell S. Methods for obtaining unpublished data. *Cochrane database of systematic reviews (Online). *Nov 9 2011(11):MR000027.

# Peer review: Referee response for version 1

**DOI:** 10.5256/f1000research.16402.r34855

**Published:** 2018-06-21

**Authors:** Harpal Uppal

**Affiliations:** 1 Lister Hospital, Stevenage, UK; 2 East and North Hertfordshire NHS Trust, Hertfordshire, UK

This interesting article systematically reviews the literature base regarding injuries of the thumb ulnar collateral ligament. A large number of studies (158) were read and assessed to identify 6 eligible comparative studies. The 6 studies in question appear to be highly heterogeneous and have a high risk of being susceptible to bias.

 The quality of published data is too poor for the study to achieve its initial goal of performing a meta analysis. This is in itself a valuable piece of information which can be used to help drive further research. 

 The search strategy is well described and repeatable and conducted according to the PRISMA checklist. 

 Overall this is a well written paper, of publishable standard, describing poor quality data which is of particular value to researchers planning new studies investigating or designing new studies concerning the ulnar collateral ligament.

I have read this submission. I believe that I have an appropriate level of expertise to confirm that it is of an acceptable scientific standard.
